# Progress Evaluation for Transnational Restaurant Chains to Reformulate Products and Standardize Portions to Meet Healthy Dietary Guidelines and Reduce Obesity and Non-Communicable Disease Risks, 2000–2018: A Scoping and Systematic Review to Inform Policy

**DOI:** 10.3390/ijerph16152732

**Published:** 2019-07-31

**Authors:** Vivica Kraak, Sofia Rincón-Gallardo Patiño, Deepthi Renukuntla, Eojina Kim

**Affiliations:** 1 Department of Human Nutrition, Foods, and Exercise, Virginia Tech, Blacksburg, VA 24061, USA; 2 Liberty University, Lynchburg, VA 24515, USA; 3 Department of Hospitality and Tourism Management, Pamplin College of Business, Virginia Tech, Blacksburg, VA 24061, USA

**Keywords:** transnational restaurants, food and beverage, product profiles, portions, healthy diet

## Abstract

Transnational restaurant chains sell food and beverage products in 75 to 139 countries worldwide linked to obesity and non-communicable diseases (NCDs). This study examined whether transnational restaurant chains reformulated products and standardized portions aligned with healthy dietary guidelines and criteria. Firstly, we describe the transnational restaurant industry structure and eating trends. Secondly, we summarize results from a scoping review of healthy dietary guidelines for restaurants. Thirdly, we describe a systematic review of five electronic databases (2000–2018) to identify studies on nutrient profile and portion size changes made by transnational restaurants over 18 years. We used Preferred Reporting Items for Systematic Reviews and Meta-Analysis (PRISMA) guidelines, identified 179 records, and included 50 studies conducted in 30 countries across six regions. The scoping review found a few expert-recommended targets for restaurants to improve offerings, but no internationally accepted standard for portions or serving sizes. The systematic review results showed no standardized assessment methods or metrics to evaluate transnational chain restaurants’ practices to improve menu offerings. There was wide variation within and across countries, regions, firms, and chains to reduce energy, saturated and trans fats, sodium, and standardized portions. These results may inform future research and encourage transnational chain restaurants to offer healthy product profiles and standardized portions to reduce obesity and NCD risks worldwide.

## 1. Introduction

Quick-service, fast-casual, and full-service restaurant (QSR, FCR, and FSR) chains offer a variety of inexpensive and convenient food and beverage products to customers on the premises, as takeaway, or delivered at home, work, or other locations [1]. Fast-food meals and sugary beverages marketed by transnational QSR chains are perceived to be a symbol of Western culture, capitalism, and the globalization of the food supply [2,3].

Technical innovation and trade liberalization enabled the international expansion of restaurant franchise businesses worldwide since the 1960s that generated some economic benefits but also many health consequences [2,3,4]. While QSR chains contributed to economic development by creating jobs, businesses often pay workers below the minimum wage and provide limited benefits that precipitate food and economic insecurity [2,4,5]. Transnational restaurant chains created a lucrative global market demand for energy-dense, nutrient-poor processed food and beverage products linked to obesity and non-communicable diseases (NCDs), including type 2 diabetes, cardiovascular diseases, and certain cancers [2,3,6]. Cross-sectional studies found associations among the consumption of food and beverage products high in energy (kilocalories (kcal) or kilojoules (kJ)) fats (i.e., total, saturated, and trans fats); sodium and added sugars; poor diet quality; unhealthy lifestyle behaviors; and obesity or diet-related NCD risks among populations in high- and middle-income countries [7,8,9,10,11,12,13,14,15].

Some evidence suggests that one’s biological preferences for energy-dense and nutrient-poor foods high in calories, fats, sugars, and sodium may interact with cultural, economic, lifestyle, and sociodemographic factors (i.e., age, race, ethnicity, and gender) to increase the demand for QSR foods [16]. Yet, two recent systematic reviews found no statistically significant association among adults’ geographic access to fast-food or QSR chains, socioeconomic status, and weight [17,18].

There is inconsistent evidence to show a strong association but not causal outcomes between fast-food or QSR consumption frequency, diet quality, and metabolic indicators of disease risk [19]. These inconsistencies may reflect differences in study design including how restaurant products are defined, the frequency and amount of food and beverage products consumed away from home, and the diet and health outcomes examined. For example, a longitudinal study of United States (U.S.) adults (1987–2017) found that, while QSR customers purchased fewer vegetables, fish, or seafood, the diet quality of QSR customers did not differ significantly from non-QSR customers [20]. A prospective cross-sectional cohort study of US adults (*n* = 3031) over 15 years (1985–2000) found that the frequency of fast-food consumption was strongly correlated with weight gain and insulin resistance [21]. However, a separate prospective cross-sectional cohort study of US adults (*n* = 9107) over 10 years (1999–2011) did not show a statistically significant association between QSR consumption frequency, cardio-metabolic risk factors, and increased mortality rates [22].

### 1.1. Study Purpose

QSR chains have extensive reach through the globalized food system and may influence the diet quality of billions of people worldwide who purchase and consume restaurant products onsite through self-serve or full-serve options, takeaway, or delivered to their worksite or home. Monitoring temporal trends in the global food supply quality, and the fidelity of QSR chain pledges and business practices over time may help to reformulate products that contribute to poor diet quality, obesity, and diet-related NCDs [23]. Documenting differences across countries and regions is important to inform government policy and hold the restaurant industry accountable for business practices to support healthy dietary guidelines and positive health outcomes for populations [24].

This study has four objectives. The first objective is to describe the transnational restaurant industry structure, global presence, and QSR consumption trends for customers. The second objective is to conduct a scoping review to identify recommendations issued by the World Health Organization (WHO) and other authoritative bodies relevant to the transnational restaurant sector to reformulate products to meet nutrient targets and standardize meal portions that align with healthy dietary guidelines. The third objective is to conduct a systematic review of peer-reviewed studies between 2000 and 2018 to understand the temporal trends in transnational chains’ reformulation of products and standardization of meal portions to align with healthy dietary guidelines. The fourth objective is to use the findings to suggest actions for future government policies and business practices for transnational QSR chains to support healthy dietary guidelines across countries and regions worldwide.

### 1.2. Restaurant Industry Structure and Global Presence

The transnational QSR chain sector emerged in the U.S. during the 1950s due to many factors including the development of new food technology, rising disposable income of households, changing work and family lifestyles, a new motorized travel infrastructure, and a growing market demand for convenient, tasty, and inexpensive food and beverage products [2,3,4]. *The McDonaldization of Society* described a “process by which fast-food restaurant businesses dominated the sectors of American society and the rest of the world” based on the principles of efficiency, calculability, predictability, and control [2].

During the 1960s and 1970s, trade liberalization and government deregulation of international markets enabled US-headquartered companies to promote branded QSR chains (i.e., McDonald’s, Subway, Kentucky Fried Chicken (KFC), Pizza Hut, Taco Bell, Domino’s Pizza, and Burger King) that used a franchising business model to increase their competitiveness by expanding rapidly into international markets [2,3,25,26]. Franchising allows a restaurant chain or *franchisor* to contract with a *franchisee* who may be an investment firm or individual who pays an initial fee (U.S. dollars (USD) $15,000–$90,000) and royalties (4% to 8% of gross sales) based on the percentage of the total annual business revenue [27,28]. The franchisee provides a location, trains employees, develops a marketing plan, and adheres to the corporate brand’s standards [27].

During the 1980s, transnational and national food and beverage manufacturers in Europe and Latin America acquired transnational QSR chains through franchising that enabled the vertical integration of processed food and beverage products into national and regional food supply chains and procurement systems that led to economies of scale to maximize sales and revenue for manufacturers [29]. Over four decades, there was a marked growth and differentiation of the restaurant industry sector in the US and worldwide, ranging from independent non-chain restaurants to the limited-service restaurant segment including QSR and FCR chains that serve burgers, sandwiches, pizza, and chicken, as well as FSR or sit-down restaurant chains, and independent family dining, casual dining, and upscale fine-dining restaurants [27,30].

Euromonitor International forecasts that the QSR chain industry will grow in most countries worldwide through 2020 [31], especially in the Asia Pacific region and China, due to the increased disposable income of young consumers who are expected to drive fast-food purchasing and consumption trends [31]. Media stories also suggested that transnational QSR chains including McDonald’s, KFC, and Domino’s Pizza expanded rapidly across Africa, Asia, and Latin America [32,33,34]. The away-from-home food sector in low- and middle-income countries (LMICs) is complex because it is comprised of both transnational and national chains, and many informal food service providers, including small-scale takeaways and street vendors [29,31]. Epidemiologic studies suggest that indigenous non-chain restaurants, independent takeaway eateries, and street vendors dominate over transnational QSR chains in India, Mexico, Nigeria, and Singapore [35,36,37,38].

Market research suggests that the global fast-food restaurant industry market represents about USD $651 billion annually [39]. By comparison, the combined U.S. limited-service restaurant (i.e., QSR and FCR) and FSR segments represented USD $497 billion and 48% of U.S. household income spent on food in 2017 [1]. Table 1 summarizes the 2018 brand value, and the number of franchise units in countries where U.S.-headquartered, popular transnational QSR chains operate businesses worldwide [40,41,42,43,44,45].

### 1.3. Customer Purchasing and Eating Trends at Restaurants

A 2015 Nielsen global online survey of more than 30,000 adults in 61 countries found that more than 50% of respondents reported eating lunch and dinner at restaurants or street vendors weekly, and about 9% ate at these away-from-home outlets daily [46]. Other research suggests that 28–37% of residents in Australia, Europe, United Kingdom (UK), and US reported consuming takeaway meals at least twice weekly [19]. Market research shows that customers who visit QSR chains rank cost, value for money spent, food quality, food safety, staff friendliness, service speed, cleanliness, atmosphere, and type of cuisine as more important attributes over personal health [47,48]. In China, consumer demand for QSR chain meals increased from USD $10.5 million to USD $94.2 million between 1999 and 2013 [49]. U.S. spending on away-from-home food accounted for 44% of total food expenditures annually since the 1980s, and increased to 50.2% in 2010 [20]. Nevertheless, customers recognize that healthy restaurant menu choices are limited. An online survey of 5000 adults across 10 high-income countries found that less than 20% were satisfied with healthy restaurant menu options [50].

## 2. Materials and Methods

This paper updates evidence from two published reviews that identified expert recommendations and evaluated the US restaurant sector progress across eight marketing-mix and choice-architecture strategies (i.e., place, profile, portion, pricing, promotion, healthy default picks, priming or promotion, and proximity) [51,52]. Given the breadth of the literature on restaurants, the research question for this study examined the use of two strategies—changing the nutrient composition by reformulating products (profiles) and reducing or standardizing serving sizes (portions)—by transnational chain restaurants worldwide to meet healthy dietary guidelines.

We defined changes to product profiles as alterations to the nutrient composition, texture, taste, and flavor of food, beverage, and meal products sold. We defined changes to portions as reducing or standardizing the food, beverage, and/or meals to influence customers’ expectations about single servings and to support healthy dietary guidelines.

### 2.1. Search Strategy for Steps 1 and 2

The first step involved the lead investigator (V.K.) conducting a scoping review of the peer-reviewed and gray-literature sources to identify dietary recommendations issued by the WHO for individuals and populations to limit nutrients of concern to reduce obesity and NCD risks. This step also involved the lead investigator contacting international researchers to identify resources that offered recommendations issued by authoritative bodies for transnational QSR chains to implement nutrient-profiling criteria or specific performance metrics for product reformulation, and portion size targets for meals, side dishes, desserts, and beverages sold to children, teens, or adults that align with a healthy diet. The results were independently reviewed by three co-investigators (S.R.G.P., D.R., and E.K.).

The second step involved two co-investigators (S.R.G.P. and D.R.) developing the search strategy, in consultation with the lead author (V.K.) and a health sciences librarian. We compiled the search terms and identified five electronic databases to search for original, peer-reviewed publications. This study’s research question was as follows: *What progress was made by transnational restaurant chains to reformulate products and standardize or reduce portions or serving sizes to meet recommended healthy dietary guidelines for children, adolescents, and adults between 2000 and 2018?*

We used the Preferred Reporting Items for Systematic Reviews and Meta-Analysis (PRISMA) guidelines [53] to conduct a systematic review of English-language electronic databases (i.e., the Cumulative Index to Nursing and Allied Health Literature (CINAHL), Food Science Technology Abstracts, Mintel, PubMed, and Web of Science) to identify relevant literature published between 1 January 2000 and 18 December 2018. Table 2 summarizes the a priori inclusion and exclusion criteria used to conduct the systematic review using the PICO approach including *population* (restaurants), *indicator* (assessment of nutrient content and portion or serving sizes of restaurant products), *comparison* (dietary standards, guidelines or nutrient profiling targets), *outcomes* (energy, fat, sugar, sodium, portion or serving size), and study design. Table S1 (Supplementary Materials) describes the detailed search strategy used for each database to conduct the systematic review. The search strategy used a combination of keywords including “restaurant”, “fast food”, “takeaway”, “take out”, “reformulation”, “change”, “portion size”, “standard”, “standardized”, “serving”, “guideline”, “diet”, “dietary”, “recommendation”, “food policy”, “nutrition policy”, “regulation”, “standard”, “monitor”, “energy”, “calorie”, “sodium”, “salt”, “saturated fat”, “trans fat”, “sugar”, “portion” and “serving”.

### 2.2. Study Selection and Data Extraction

One co-investigator (S.R.G.P.) removed duplicate articles from the original search of the five electronic databases. Two co-investigators (S.R.G.P. and D.R.) screened the titles and abstracts of the retrieved records independently to identify studies that met the inclusion criteria, as recommended by the PRISMA process. A third co-investigator (V.K.) confirmed the inclusion or exclusion of the retrieved records. The three co-investigators discussed any disagreements, and documented reasons for study exclusions that included location (i.e., conducted in other settings such as schools, childcare, cafeterias, supermarkets, vending machines, or convenience stores), and marketing practices (i.e., television, internet, outdoors, or mobile devices) that were not the focus of this study. Two co-investigators (S.R.G.P. and D.R.) examined the full-text key review articles included, and hand-searched the references of selected articles and key review articles. They consulted other sources to ensure that they had not overlooked any important articles published during the review period. One co-investigator (D.R.) extracted relevant data from each study verified independently by two co-investigators (V.K. and E.K.). The evidence table included the lead author and year the study was published, study objective, data collection period, study design and methods (i.e., outcomes measured, assessment, evidence sources, and dietary or nutrient-profile guidelines or criteria used to assess the nutrient composition or serving size of products), restaurant chains examined, and the main results.

### 2.3. Study Quality Assessment

Two co-investigators (S.R.G.P. and E.K.) independently reviewed the 50 studies for quality. They used the Johanna Briggs Institute’s eight-item, critical appraisal checklist for analytical cross-sectional studies [54] to assess the quality of each study for clear inclusion criteria, setting, measuring the exposure in a valid and reliable way, and whether objective and standard criteria were used to measure the outcomes of interest. Each study was assigned a quality score of weak (1), moderate (2), or strong (3). A third co-investigator (V.K.) resolved differences in scoring, and consensus was reached on the final score through investigator triangulation. We did not conduct a risk of bias assessment because the outcomes of interest were descriptive, and the systematic review did not include either intervention or randomized controlled trial study designs.

## 3. Results

The section below describes the results for step 1 (scoping review) and step 2 (systematic review) based on the findings from published studies presented in a narrative summary. The different study designs and heterogeneity of study outcomes precluded the poling of data to conduct a meta-analysis for the results across countries.

### 3.1. Step 1: Scoping Review of Dietary Recommendations for Restaurant Chains

More than 100 countries worldwide developed science-based, national dietary guidelines to inform food and nutrition policies for stakeholders to foster a healthy diet [55]. The WHO and Food and Agriculture Organization (FAO) of the United Nations issued several reports between 2004 and 2018 with recommendations for national governments to increase nutrient density of diets and reduce several nutrients of concern in the food supply. Recommendations for individuals and populations to consume nutrient-dense foods include five or more servings (more than 400 g) daily of fruits and vegetables [56]; lean meat and fish, low-fat or fat-free dairy, or appropriate plant-based substitutes; dietary fiber (20 g/person/day) [57,58]; and dietary potassium (e.g., beans, peas, nuts, and fruits and vegetables) to provide at least 3.5 g potassium/person/day [59].

The WHO and/or FAO also recommended that individuals and populations reduce dietary fats (i.e., total, saturated fat and artificial or industrially produced trans fats (TFA)), and replace TFA with healthier monounsaturated or polyunsaturated fats or oils [60,61]. Additionally, individuals should reduce free or added sugars [62] and sodium or salt [63] to lower obesity and diet-related NCD risks including type 2 diabetes. The specific targets recommended are as follows: dietary fat (15–30% total energy/person/day); saturated fat (<10% total energy/person/day; <7% total energy for high-risk groups); and TFA (<1% of total energy intake that translates into <2.2 g TFA/day for a 2000-calorie diet (one kilocalorie (kcal) = 4.184 kilojoules (kJ); a 2000-calorie diet is equivalent to an 8370-kilojoule diet). Moreover, the WHO recommended that consumers reduce their free or added sugars to less than 10% total energy/person/day, representing 25 g/day or six teaspoons added sugars for children, and 50 g/day or 12 teaspoons added sugars for adults who consume a 2000-calorie diet [62]. Finally, individuals should aim to consume less than a teaspoon of salt (less than 5 g salt/person/day) or sodium (less than 2 g sodium/person/day) [63].

Several national governments issued dietary recommendations for the average adult to consume no more than 2000 to 2500 kcal or 8700 kJ daily [64,65,66,67]. In 2018, Public Health England launched the One You Campaign that encouraged adults to “Aim for 400-600-600” when eating away from home, by choosing 400 calories for breakfast, 600 calories for lunch, and 600 calories for the dinner meal [68].

Governments, industry task forces, and public health experts in Australia [69], Canada [70], the United Kingdom [71], and the United States of America (USA) [72,73] issued recommendations for healthy food procurement and nutrition standards for various food service settings. However, there are few explicit recommendations for transnational chain restaurants with quantitative nutrient targets and specified timelines to improve the healthfulness of offerings. Moreover, there is no internationally accepted standard for a portion or serving size of a meal for a child, adolescent, or adult. Table 3 summarizes the recommended dietary guidelines identified through the scoping review from bodies that issued specific dietary recommendations, nutrient targets, or performance metrics for restaurant chains to reformulate products and reduce or standardize meal portions to support healthy dietary guidelines [74,75,76,77,78]. Only the U.S. National Salt Reduction Initiative offered a specific timeline for restaurants to implement the sodium recommendations [78].

### 3.2. Step 2: Data Selection, Quality Assessment, and Analysis for the Systematic Review

**Figure 1:**
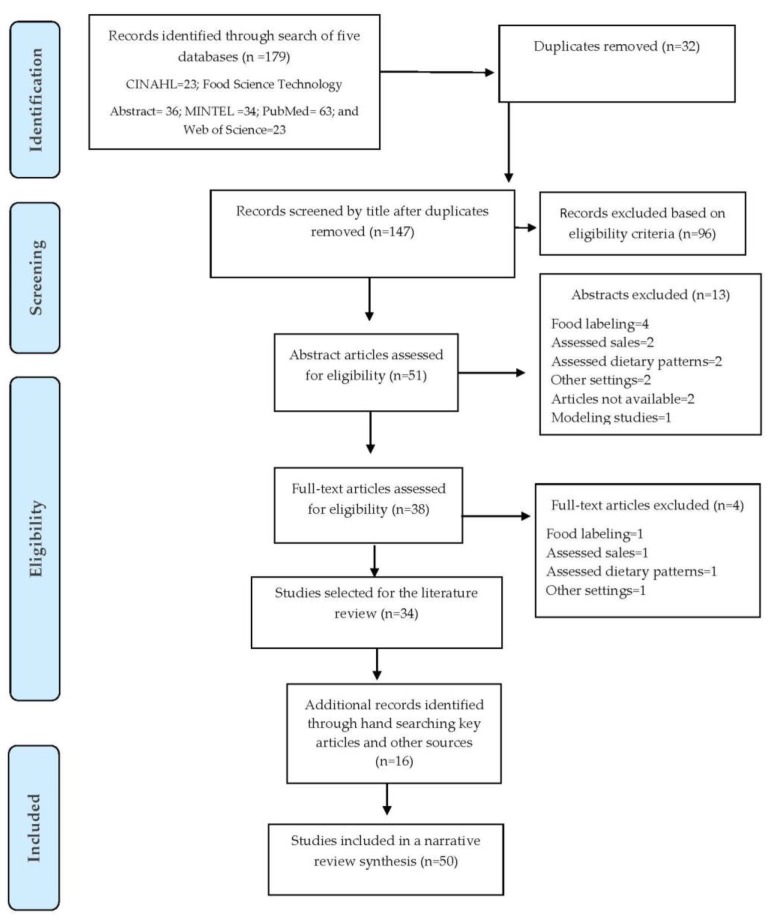
Preferred Reporting Items for Systematic Reviews and Meta-Analysis (PRISMA) flow diagram for the systematic review of studies to evaluate transnational restaurant chains’ progress to reformulate products and standardize portions to meet healthy dietary guidelines, 2000–2018.

Figure 1 shows the PRISMA flow diagram that shows that the initial search yielded 179 records from five electronic databases. After removal of duplicates (*n* = 32), 147 titles and 51 abstracts were screened. Following the full-text review (*n* = 38), we excluded four studies and selected 34 studies that met the inclusion criteria. An additional 16 additional articles were identified from the reference lists of key articles and other sources, and 50 articles were included in the final analysis [78,79,80,81,82,83,84,85,86,87,88,89,90,91,92,93,94,95,96,97,98,99,100,101,102,103,104,105,106,107,108,109,110,111,112,113,114,115,116,117,118,119,120,121,122,123,124,125,126,127].

Table 4 summarizes the Johanna Briggs Institute critical appraisal checklist for quality assessment results for eight items that rated the study quality as weak (1), moderate (2), or strong (3). This tool examined whether the study had clearly defined inclusion criteria, the detail described for the study setting, validity and reliability of the outcomes measured, use of standard criteria to measure outcomes, strategies to address confounding factors, and statistical analysis used. 

### 3.3. Study Characteristics: Outcomes, Research Design, Duration, Restaurant Segment, Geographic Location

Table 5 summarizes the studies included in the systematic review by country, study design, methods, data collection period, outcomes measured, and number of chains examined. A majority of studies (*n* = 39) were cross-sectional and 11 studies were longitudinal. The studies compared outcomes of interest over several weeks to years between 2000 and 2018. The outcomes measured were energy (kcal/kJ) or energy density (mg/100 g) (*n* = 36 studies); total, saturated, and TFA (g)) (*n* = 35 studies); free or added sugars (g) (*n* = 13 studies); sodium or salt (mg) or sodium density (mg/1000 kcal or 1000 kJ) (*n* = 34 studies); and portion or serving size (kcal or kJ/serving or per 100 g) (*n* = 9 studies). Table S2 (Supplementary Materials) summarizes the detailed findings for the published studies included in the systematic review, including the lead author and year published; study purpose; location (continent, country and state); data collection period; study design; assessment methods and evidence sources; number and type of restaurant chains examined; and main results.

Of the 50 studies summarized in Table 5 and Table S2 (Supplementary Materials), 30 studies reported examining 475 chains from the QSR, FCR, and FSR segments. McDonald’s Corporation was reported at the highest frequency of studies (*n* = 25), followed by Burger King or Hungry Jack’s (*n* = 24), KFC (*n* = 22), Subway (*n* = 16), Pizza Hut (*n* = 13), Domino’s Pizza (*n* = 10), and Taco Bell (*n* = 6). The remaining 20 studies did not report the QSR, FCR, and FSR chains and non-chain restaurants. Only three studies conducted in Abu Dhabi, United Arab Emirates (UAE); London, England, United Kingdom (UK); and Japan [95,107,120] compared the nutritional profile and/or portion sizes of items at chain restaurants and non-chain restaurants.

Table S3 (Supplementary Materials) lists 50 studies conducted in 30 unique countries and six geographic regions (i.e., Africa, Americas, Asia, Europe, Middle East, and Oceania). Most studies were conducted in one country. Six multi-country studies [91,98,101,119,127] compared specific outcomes of interest for restaurant chains in 48 countries between two and five geographical regions from 2006 to 2018. More than half of the studies were conducted in the North American region including the US (*n* = 29) and Canada (*n* = 9), followed by Oceania that included Australia (*n* = 9) and New Zealand (*n* = 7). Three studies examined QSR outcomes in Africa including in Egypt (*n* = 1), Ghana (*n* = 1), and South Africa (*n* = 1). Four studies were conducted in Latin or South America including Brazil (*n* = 1), Costa Rica (*n* = 1), Guatemala (*n* = 1), and Peru (*n* = 1). Six studies were conducted in Asia including Japan (*n* = 2), China (*n* = 2), and India (*n* = 2). In the European region, the United Kingdom (England) (*n* = 5) was followed by one or more studies conducted in Austria, Czech Republic, Denmark, Finland, France, Germany, Hungary, Italy, the Netherlands, Norway, Poland, Portugal, Russia, Spain, and Sweden. Two studies were conducted in the UAE in the Middle East region. Fifteen studies [78,89,92,95,98,101,102,103,104,105,107,113,114,120,124] evaluated children’s menu items across six regions for eight countries including Australia, Canada, Guatemala, Japan, New Zealand, UAE, UK, and U.S.

### 3.4. Study Characteristics by Evidence Sources, Assessment Methods, and Dietary Guidelines or Criteria

Several studies used one or more forms of primary, secondary, or tertiary information, diverse assessment methods, and statistical tests to determine whether there were significant changes in the nutrient content or portion size changes of menu items over time. These methods included the annual tracking of restaurant products reported by manufacturers on their websites, onsite visits to examine offerings on menus and menu boards, and by contacting restaurant firms via telephone. A few studies collected menu samples (i.e., fries, hamburgers, or chicken dishes) homogenized and tested in the laboratory using bomb calorimetry to measure energy or gas chromatography to measure the TFA content of restaurant menu items compared within and across countries [80,108,119]. Nine U.S. studies used the MenuStat Database established by the New York City Department of Health and Mental Hygiene to provide nutrition information for products sold by the leading QSR, FCR, and FSR chains from 2012 to 2017 [73,83,84,85,92,100,104,110,126]. Four studies used independent food and nutrient databases to assess the quality of meals [82,96,109,115].

Different dietary guidelines were used in the studies that varied by study design and country. About two-thirds (*n* = 34/50; 68%) of the studies reported guidelines, criteria, or nutrient targets to assess the healthfulness of menu offerings based on comparative standards for the product profiles or portion sizes. Three of the five multi-country studies [91,98,127] did not report any standard dietary criteria to compare differences across countries or regions (Table S3, Supplementary Materials).

The US and a few non-U.S. studies reported using the Dietary Guidelines for Americans (DGA) 2005, 2010, or 2010–2015 or the percent dietary reference value (%DV) for selected nutrients [79,81,87,89,92,96,99,101,102,112,116,117,126], the FDA or American Heart Association’s sodium targets [79,99], The U.S. Department of Agriculture’s (USDA’s) Healthy Eating Index [79,96,102], National School Lunch Program (NSLP) standards [95,104], US Expert Panel on Children’s Menu Portions [78], the National Restaurant Association’s Kids LiveWell Program [95,104], and the American Academy of Pediatrics’ energy recommendations [92] (Table 3). Other studies reported using the Dietary Guidelines for Children and Adolescents in Australia [124], Japanese NSLP standards [120], UK’s Nutrient Profiling Model [103,118], Recommended Dietary Intakes of New Zealand [123], and the WHO target for sugars [123].

### 3.5. Energy

Thirty-six studies measured energy (kcal/kJ) either as a primary outcome or to calculate the energy density for fat, TFA, sugar, or sodium content/1000 calories. Fewer studies measured either total fat, saturated fat, and/or TFA (*n* = 28); free or added sugars (*n* = 12); and portion or serving size (*n* = 19) reported as kcal/kJ or energy density/100 g. A specific outcome for energy at restaurant chains was reported for 33 studies conducted in eight countries including Australia, Canada, Guatemala, Japan, New Zealand, UAE, UK, and the U.S. Four multi-country studies [98,101,108,127] compared the energy and/or portion sizes at chain and non-chain restaurants collectively across 24 countries, and all documented considerable variability in energy (kcal/kJ) and fat content across different menu items, chain types, and countries. More than one-third (38%) of 36 studies examined the energy content of children’s menu items, including one multi-country study that compared the energy content of QSR menu items in Australia, Canada, New Zealand, UK, and U.S. [98]; six U.S. studies [78,92,102,104,105,117], and one study each in Australia [124], Canada [113], Guatemala [103], Japan [120], UAE [95], and the UK [107].

The diversity of dietary guidelines and nutrient targets used, the examination of different chain types (i.e., QSR, FCR, and FSR), menu items (i.e., bundled meals, entrees, burgers, fries, side dishes, and beverages), varied age groups (i.e., adult versus children), and time frame from 2000 to 2018 precluded making direct comparisons of the energy content of chain restaurants’ menu items across the 33 studies. Therefore, we provide a narrative summary of salient results for energy outcomes below discussed chronologically for each of the eight countries.

In Australia, Brindal et al. (2008) [86] documented that the average meal provided nearly half (47.5%) of energy (kJ) and fat (g) (48%) at six chains in 2005. By 2009, Dunford et al. (2010) [90] found that a majority of items examined at nine chains did not meet healthy criteria. In 2010, Wellard et al. (2012) [124] examined the nutrient content of 199 children’s meal combinations and found that only 16% and 22% met the industry’s nutrient criteria for children aged 4–8 and 9–13 years, respectively. More than two-thirds (72%) of QSR meals exceeded 30% of the daily energy recommendations for a four-year-old child, and many meals also exceeded the upper limit for daily saturated fat recommendation for children aged 4–8 years. Between 2009 and 2015, Wellard-Cole et al. (2018) [125] found that five chains significantly increased the energy content for limited-time menu offerings over seven years despite voluntary menu labeling legislated in New South Wales, Australia.

In Canada, Scourboutakos and L’Abbe (2012) [111] documented that FSR chains had higher calories/serving for all food categories compared to QSR chains in 2010. Calories varied both within and across food categories, and the portion or serving size was more strongly correlated with calories than energy density at 85 chains examined. Between 2010 and 2011, Scourboutakos et al. (2013) [112] documented that, among 19 FSR chains, meals provided an average of 1128 calories (56% of daily 2000 calories/day), 89% DV for fat, and 83% DV for saturated fat; these restaurants labeled meals as healthy if they provided an average of 474 calories, 13 g fat, and 3 g saturated fat/serving. Scourboutakos et al. (2014) [113] also found that half (50%) of children’s meals sold at 17 chains exceeded the WHO’s daily free sugars target (5–10% energy) in 2010.

In New Zealand, Chand et al. (2012) [88] found that only one-fifth (21%) of items met healthy guidelines in 2010–2011, and 79% of items were high in energy and exceeded the portion size target at 12 chains. In 2014, Waterlander et al. (2014) [123] found that the most popular burger combo meals and pizza sold at four chains contributed between one-third and half of an adult’s energy needs, and the combo meals provided at least 94% of the WHO’s free sugars guideline. From 2012–2016, Eyles et al. (2018) [93] documented moderate to large increase in mean portion size and energy density for all menu items examined at 10 QSR chains.

In the UK, Reeves et al. (2011) [107] examined the mean portion size of children’s meals at seven chain and non-chain restaurants in 2009 and found that QSR chains provided smaller portions compared to non-chain FSR; however, neither the QSR nor FSR meals met the recommended nutrient standards for children aged 5–11 years. In Guatemala, Mazariegos et al. (2016) [103] found that six chains marketed less than one-fifth (18.4%) of combination meals in 2016, but none of the five children’s meals that provided nutritional information met healthy dietary guidelines. In the UAE, Garemo and Naimi (2018) [95] documented that half of 58 chain and non-chain restaurants offered children’s menus in 2016, but more than three-quarters (79%) of these meals did not meet the US Kids LiveWell Program healthy criteria. In Japan, Uechi 2018 [120] examined the nutritional content of children’s meals at 20 chains in 2017 and found that more than half of the restaurants aligned with the nutrient standards of the Japanese School Lunch Program for energy. Overall, about 59% and 41% of children’s meals met the energy (≤2218 kJ) and fat (≤30% energy) content, respectively.

In the US, a majority of the 19 studies that examined the quality of menu items documented that most menu entrées or bundled meals sold to adults exceeded 700 kcal/meal [81,108], and received a low Healthy Eating Index score [96,102,105]. Selected results for the U.S. studies are presented below.

Between 2000 and 2013, Urban et al. (2014) [121] documented that the energy content of 56% of items decreased and the energy content of 44% of items increased at three chains. In 2013, energy content of a large-sized bundled meal (cheeseburger, fries, and soda) represented 65–80% of a 2000 calorie diet. Between 2006 and 2010, Bauer et al. (2012) [82] found no change in the energy for entrees and beverages, and energy for side dishes decreased but desserts increased at eight chains. 

From 2009–2010, Bruemmer et al. (2012) [87] documented a lower energy content for items at 37 FSR and LSR chains, yet all chains exceeded the DGA 2005 for calories (56%) and saturated fat (77%). Soo et al. (2018) [118] found that the promoted items on the general menu boards in 2010 and 2013 did not meet healthy nutrient criteria at four U.S. chains.

Two of six U.S. studies assessed children’s meals between 2008 and 2009 including O’Donnell et al. (2008) [105], which documented that only 3% of meals met all NSLP nutrition criteria at 10 chains, and non-adhering meals were more than 1.5 times energy dense than healthy meals; and from 2008–2009, Kirkpatrick et al. (2013) [102] observed that the menus at five QSR chains scored lower than 50/100 on Healthy Eating Index 2005, although children’s menus scored 10 points higher than adult meals. However, no menu received a score higher than 72 out of 100 points. Meal scores for total fruit, whole grains, and sodium were poor. Between 2012 and 2016, four studies of children’s meals documented that, while QSR chains were more likely to provide healthier options than FSR chains, most menu items did not meet the DGA targets for calories, or percent calories from fat or saturated fat, and FSR chains were more likely to serve children’s meals that exceeded ≤600 calories/meal [78,92,117]. Moran et al. (2017) [104] found that, between 2012 and 2015, 15 out of 45 chains that participated in the US Kids LiveWell Program significantly reduced the energy by 40 calories/children’s meal compared to nonparticipating restaurants, but this change did not persist through 2015.

Several U.S. studies used the New York City MenuStat Database to examine trends in the energy content of chain restaurant menu items from 2012 to 2015 [83,84,85,100,110]. Bleich et al. (2015) [83] observed a modest decline in energy (8–20%) for newly introduced entrees, beverages, and children’s meals but no differences in mean calories for menu items at 66 chains. Jarlenski et al. (2016) [100] found a modest decline in energy across 11,737 items, but an increase in energy of beverages and a large percentage of calories from added sugars in desserts at 37 chains. Bleich et al. (2017) [85] found no differences in mean energy for newly introduced items (2012–2015) relative to items on menu in 2008 at 44 chains. Schoffman et al. (2016) [110] found that FCR chains provided more calories per entrée (760 kcal) than QSR entrées (561 kcal), and QSRs provided more entrées <500 calories compared to FCR chains >751 calories at 62 chains.

### 3.6. Fat, Saturated Fat, and TFA

Of the 26 studies that examined changes in dietary fats, the results suggest a reduction in TFA to approach recommended levels over 18 years, but not a reduction in total or saturated fats of other food items. Auchincloss et al. (2014) [81] documented in 2011 that 30% of à la carte entrees at US chains exceeded the percent DV for saturated fat. In 2004 and 2005, Stender et al. (2006) [119] tested and compared two menu items (i.e., fries and chicken nuggets) at McDonald’s and KFC across 20 countries and three regions. These investigators found that half of the 43 samples contained >5.0 g TFA/serving, with KFC providing more TFA/item serving compared to McDonald’s. The investigators also found wide variation in the TFA content of items across the two chains depending on the chain’s geographic location ranging from 1–2% TFA in Denmark, 1–13% TFA in Spain, 5–23% TFA in the U.S., and 8–35% TFA in Hungary. By 2010–2011, Scourboutakos et al. (2013) [112] documented that meals sold at 19 FSR chains in Canada provided an average of 0.6 g TFA/meal.

By 2017, Astiasarán et al. (2017) [80] documented fries had a TFA content ranging from 0.49% to 0.89%, which was lower than <2% total energy set by European countries as the maximum legal content of TFA that contained <0.5 g/serving. In the U.S., Urban et al. (2014) [122] documented a sharp decline in saturated fat and TFA of large fries/1000 calories at three chains. After 2009, cheeseburgers were the major contributor of TFA/1000 calories, and the TFA content of this item remained stable through 2013. Finally, two studies of children’s meals reported on the TFA content. In 2012–2014, Eissa et al. (2017) [92] found that the TFA content in children’s meals was lower at QSR chains compared to FSR chains based on an analysis of 42 chains. In 2016, Mazariegos et al. (2016) [103] reported zero TFA content of five children’s meals at six chains that provided nutrition information in Guatemala.

### 3.7. Sodium or Salt

Of the 38 studies that examined the sodium or salt content of chain restaurant meals, two multi-country studies documented substantial differences in the sodium content of menu items that differed by food category at different QSR chains across nine countries [91,101]. Dunford et al. (2012) [91] documented that the mean sodium content of foods varied between chains and between the same products across six countries, and Khan et al. (2018) [101] found that two-thirds of sodium came from meats, chicken, and buns across four countries including Australia, Egypt, India, and the U.S. Studies across Australia [94,124], Canada [113,114,115,116], Costa Rica [97], Japan [120], New Zealand [93,106,123], and the U.S. [79,87,89,99,109,121,122,126] documented wide variation in the sodium content by restaurant chain and food category that precluded making generalizations about the sodium content of menu items within a single country and across countries. Only a small proportion of children’s meals met the recommended sodium targets in Australia [124], Japan [120], and U.S. [117]. A large proportion of entrees sold at U.S. chains exceeded the sodium target recommended by the DGA or FDA [79,87,89,122], and the sodium content of items either increased between 2000 and 2014 or decreased modestly for newly introduced items between 2012 and 2016 [99,109,121].

## 4. Discussion

This is the first systematic review to summarize the findings from the peer-reviewed literature to determine whether transnational restaurant chains used two strategies, reformulating food and beverage products and standardizing menu item portions and servings, to align with healthy dietary guidelines across franchise businesses in countries worldwide between 2000 and 2018. This study is important because poor diet is the leading risk factor for NCD mortality, especially among LMIC populations, associated with 11 million deaths and 255 million disability-adjusted life years [128]. A recent analysis showed an increase in the volume of ultra-processed food and beverage sales in South and Southeast Asia and North Africa and the Middle East between 2002 and 2016, which was positively associated with increased obesity risk during this period [129]. The WHO Action Plan encouraged governments and other stakeholders to adopt policies and actions to halt obesity and diabetes rates and reduce NCD mortality. Relevant actions include eliminating TFA and reducing sodium by 30% by 2025 [130].

These study results may inform decision-makers at transnational chain restaurants, and their franchise businesses to improve customers’ perceptions of their corporate brand image, increase trust, and brand loyalty [131], while also promoting healthy profile products and small portions that align with public health recommendations.

Step 1 of this study involved conducted a scoping review of dietary recommendations for restaurants chains. We identified general dietary and nutrient-specific recommendations issued by the WHO and/or FAO for individuals and populations to reduce processed foods and beverages high in energy, saturated fat, TFA, free or added sugars, and sodium. We also identified national governments and public health experts in Australia, Canada, the UK, and U.S. that issued recommendations for healthy food procurement and nutrition standards for various food service settings. We found only a few recommendations for transnational chain restaurants with quantitative nutrient targets but no clear specified timeline to improve the healthfulness of offerings.

The results of step 1 revealed a lack of clear, universal and internationally accepted standards for transnational restaurant chains to adopt portion or serving sizes for meals, beverages, side dishes, and desserts served to children, adolescents, and adults. Downsizing and standardizing portions are recommended as an important strategy for restaurants to reduce obesity and NCD risks for customers [51,132,133]. Some restaurant owners expressed concern about voluntarily reducing meal portions due to anticipated loss of revenue, lack of customer demand, and limited technical assistance [134].

A US evaluation of restaurant industry progress included four studies that found restaurant owners who participated in healthy restaurant programs were receptive to reducing portion sizes of children’s meals or side dishes, and the reductions led to fewer calories purchased or consumed by children [52]. Research also suggests that modest reformulation of restaurant products to reduce calories, fat, saturated fat, and sodium are acceptable to consumers [135].

Given the public scrutiny on transnational restaurant chain business practices that may contribute to obesity and NCDs, there is a need for an industry body, such as the US National Restaurant Association or International Food and Beverage Alliance, to encourage transnational chains to adopt standardize nutrient targets that meet healthy dietary guidelines (Table 3) and applied across all countries where they operate franchise businesses (Table 1). McDonald’s Corporation is the only transnational QSR chain that publicly announced commitments and performance metrics to sell healthy children’s meals in markets throughout Asia, Canada, Europe, and the U.S. by 2025 [136,137]. This global commitment is important for other chains to adopt because parents request that chain restaurants provide less expensive and smaller portions of healthy choices for their children [138].

Step 2 of this study involved conducting a systematic review of the peer-reviewed literature. We identified 50 published studies that collectively revealed great variation in the outcomes examined in 30 countries and five regions worldwide. A majority of studies that examined the energy and sodium content of menu items were in single countries, with only six multi-country studies that measured differences in these nutrients in 48 countries. Three-quarters (77%) of the studies were from high-income countries. Only five studies included LMICs in Africa (i.e., Ghana and Egypt) [101,108], Asia (i.e., China and India) [101,108], and Latin America (i.e., Brazil, Guatemala, and Peru) [103,108,119].

The results showed that two-thirds (68%) of the 50 studies reported various government, industry, or WHO guidelines or criteria used to compare their results. Due to the diversity of dietary guidelines and nutrient targets used, along with different menu items examined across various QSR, FCR, and FSR chains and non-chain restaurants, we were unable to directly compare the nutrient content changes in menu items within and across the countries between 2000 and 2018.

Recent U.S. studies (2012–2015) suggested that the 2010 national menu labeling law may have influenced leading chain restaurants to reformulate by reducing the energy content for newly introduced items, but not existing or time-limited menu items [83,84,85]. A separate evaluation showed that 78% of 90 popular QSR, FCR, and FSR chains either fully or partially complied with the U.S. national menu labeling law by the May 2018 implementation date [139]. However, the U.S. law does not require labeling disclosures for other nutrients of concern and thus may not have stimulated industry reformulation to reduce saturated fat, added sugars, and sodium in menu items.

The nutrient composition findings for menu items in other countries were less optimistic. In Australia, the energy and sodium content of menu items increased between 2009 and 2015 despite voluntary menu labeling legislated during this period [125]. Similarly, the body of research for Canada, New Zealand, and the UK reflected only a small proportion of adult or children’s meals that met healthy criteria. Governments could adopt policies and enact mandatory legislation to encourage the restaurant industry sector to meet healthy reformulation targets and standardize portions.

The WHO set a global target and commitment to assist governments and food service industries to eliminate industrial TFA from processed foods and use alternative healthy fat replacement by 2023 [140]. Only a few of the 28 studies reported on the TFA content of selected menu items. We found limited evidence that transnational restaurant chains reduced or eliminated TFA in menu offerings, which is especially relevant for chains that are expanding in LMIC markets.

Businesses, researchers, and civil society organizations could monitor and evaluate transnational chain restaurants’ progress to provide offerings that meet dietary guidelines and criteria across countries and regions. There is also a need for a central database to compare results across countries. An international collaboration was initiated in 2012 to track changes in the composition of fast foods across countries [141] but requires funding to support monitoring as the restaurant sector makes continuous changes in menu offerings. The Access to Nutrition Foundation monitors the nutrition-related policies and practices of global food and beverage manufacturers but not transnational restaurant chains [142]. This foundation could expand its monitoring efforts to the restaurant sector or fund researchers and civil society organizations to use the Business Impact Assessment (BIA)-Obesity [143] tool to compare policies, commitments, and actions to reformulate and reduce portions of menu items across countries and regions.

Researchers could also examine how transnational restaurant chains can combine marketing-mix and choice-architecture strategies to provide profitable and healthy options. Examples include using proportionate pricing and price promotions for healthy and affordable products; designating fruit, salad, and water as healthy default picks; using proximity or positioning to promote healthy affordable options; and using priming or prompting through information, labeling, or verbal cues to encourage customers to “downsize” portions to reduce their energy intake [51,52,144].

### Study Strengths and Limitations

Strengths of this study were the aggregate of evidence for many types of transnational chain restaurants, the range of dietary outcomes examined for various menu offerings, the examination of temporal trends over 18 years for product reformulation, and portion sizes of items sold by restaurant chains countries and regions worldwide. We also assessed the quality of the studies published that was not conducted for previous systematic reviews published. Limitations were that we focused on comparing outcomes for nutrients of concern and not desirable nutrients such as whole grains and dietary fiber. We also limited this review to examined English-language, peer-reviewed publications and did not include studies published in other languages or gray-literature evidence sources such as industry or civil society organization reports. We also did not have access to proprietary industry data that could have revealed the frequency of consumption or sales by restaurant brand in the countries and regions examined. The different study designs and heterogeneity of study outcomes precluded the pooling of data to conduct a meta-analysis for the results across countries. Future research is needed to examine restaurant chain marketing practices aimed at children, variations in pricing strategies, and use of healthy default beverage and side dish choices for customers. These topics were beyond the scope of this study but are needed to inform future government policies and business practices.

## 5. Conclusions

The results of this scoping and systematic review suggest that there are only a few expert-recommended targets for transnational chain restaurants to improve the quality and healthfulness of their offerings to customers, but no internationally accepted standard appropriate for portion or serving sizes to prevent obesity. We also found no standardized assessment methods or metrics to evaluate transnational chain restaurants’ practices to improve menu offerings. There was wide variation within and across countries, regions, firms, and chains to reduce energy, saturated and trans fats, sodium, and standardized portions. Researchers should use standardized assessment methods, tools, and performance metrics to evaluate transnational chain restaurants’ practices to improve diet quality of menu offerings across countries and regions. These results may inform future research and encourage transnational chain restaurants to offer healthy product profiles and standardized portions to reduce obesity and NCD risks worldwide.

## Figures and Tables

**Table 1:** Major United States (U.S.)-headquartered transnational quick-service restaurant (QSR) chains, 2018 brand value ^1^ and number of franchise units in countries where the businesses chains operate worldwide. KFC—Kentucky Fried Chicken; USD—United States dollars.

QSR Chain	2018 Brand Value ^1^ USD $ Billion	Franchise Units and # Countries
McDonald’s	$126.0 billion	>33,500 units in >100 countries
Starbucks	$44.5 billion	>29,000 units in 75 countries
Subway	$18.8 billion	>44,000 units in 112 countries
KFC ^2^	$15.1 billion	>45,000 units in 139 countries ^2^
Pizza Hut ^2^	$7.4 billion	
Taco Bell ^2^	$5.2 billion	
Domino’s Pizza	$7.4 billion	>11,000 units in 85 countries
Burger King ^3^	$5.1 billion	>15,000 units in >100 countries

^1^*Brand value* is the worth of a company’s total assets (i.e., financial and intangible) based on the firm’s name, design, symbol, and other features that identify its products from another firm, and represents the extra amount that customers are willing to pay over alternative brands. ^2^ KFC, Pizza Hut, and Taco Bell operate under Yum! Brands, Inc. in the U.S. but operate under other parent companies and licensees in other countries. For example, Yum China Holdings Inc. is a licensee of Yum! Brands in Mainland China. ^3^ Burger King Corporation operates as a fast-food franchise business under the corporate name Hungry Jack’s Pty Ltd., a subsidiary of Competitive Foods Australia, in Australia.

**Table 2:** Inclusion and exclusion criteria for the study selection for the systematic review. FCR—fast-casual restaurant; FSR—full-service restaurant; TFA—trans fatty acids.

PICO	Inclusion Criteria	Exclusion Criteria
**Population**	QSR, FCR, and FSR chain restaurants or firms that operated businesses in high-, middle-, or low-income countries.	Study reported on non-chain restaurants or other food service industry settings (i.e., worksites, cafeterias, canteens, schools, childcare, and supermarkets).
**Indicator**	Standardized assessment methods used to determine the nutrient content of food or beverage products, side dishes, and meals sold at restaurants.	Study did not report or use standardized assessment methods to determine the nutrient content or portion size of meal items available to customers to purchase or consume.
**Comparison**	Nutrient content and portion size of food and beverage items compared to standardized or recommended healthy dietary criteria or targets.	Study did not report nutrient-profile criteria or targets to compare product profiles or product portion or serving size related to the outcomes of interest.
**Outcomes**	Nutrient profile or composition Energy (*calories or kilojoules or energy density*); Fats (*total, saturated, and TFA*); Sugars (*total, added, or free*); and Sodium *(salt or sodium density).* Portion or serving size *Meals, non-alcoholic beverages or drinks, side dishes, desserts, or other edible products.*	Study did not report on the nutrient profile, nutrient composition, or outcomes of interest.
**Study Design**	English language, published between 1 January 2000 and 18 December 2018. Longitudinal, cross-sectional, descriptive, observational, and/or intervention studies.	Study was non-English language, published before January 2000 or after December 2018, gray-literature source, or the full record was not available for review.

**Table 3:** Recommended dietary guidelines for restaurant chains to reformulate and standardize serving sizes of products to meet healthy dietary guidelines *.

Year	Authoritative Body or Program	Recommended Food Groups, Dietary Guidelines or Nutrient Targets
2011	U.S. National Restaurant Association and Healthy Dining’s *Kids LiveWell* Program [74]	**To participate in the *Kids LiveWell* Program, a restaurant must provide at least one meal that meets the following criteria:** ≤600 calories/meal ≤35% calories from total fat/meal ≤10% calories from saturated fat/meal <0.5 g TFA/meal ≤35% calories from total sugars/meal ≤770 mg sodium/meal *Food groups:* Two or more servings of fruit, vegetables, whole grains, lean protein, and/or low-fat dairy. The meals must offer at least one other side item with ≤200 calories, ≤35% of calories from total fat, ≤10% calories from saturated fat, <0.5 g TFA, ≤35% of calories from total sugars (added and naturally occurring), ≤250 mg sodium; and at least one serving of fruit, vegetables, whole grains, lean protein and/or low-fat dairy. Restaurants may offer >½ cup of dairy; and 2% milk allowed if included in the meal that meets the overall criteria.
2012	American Heart Association’s (AHA’s) Heart Healthy Program [75]	**To participate in the AHA’s certified Heart Healthy Program, an adult meal must meet the following criteria:** ≤700 calories/meal *(a meal includes an entrée, side, and beverage)* ≤30% calories from total fat, ≤3 g total fat/100 g and ≤26 g total fat/meal ≤10% calories from saturated fat, ≤5 g saturated fat/meal ≤105 mg cholesterol/meal or ≤20 mg cholesterol/100 g/meal <0.5 g TFA/meal ≤960 mg sodium/meal No recommendation for added sugars *Beneficial nutrients*: ≥10% of daily value for at least one nutrient/meal for vitamin A, vitamin C, calcium, iron, dietary fiber (≥2.5 g/100 g), or protein (≥5 g/100 g) If the meal includes a beverage (i.e., water, fat-free or low-fat milk), it must be included in the meal profile and beverages must contain <10 calories/serving. Excluded items are alcoholic beverages, desserts, and products that do not align with the AHA’s healthy diet and lifestyle criteria.
2013	U.S. National Institutes of Health (NIH) and RAND Corporation Expert Panel for Healthy Restaurant Meal Standards [76]	**The NIH and RAND healthy restaurant criteria define a meal (i.e., entrée, side, and beverage) as meeting the following criteria:** ***Adult meals*** ≤700 calories/meal ≤10% of calories from saturated fat/meal <0.5 g of TFA/meal ≤35% calories from total sugars/meal ≤770 mg sodium/meal ≥1.5 cups of vegetables or fruits (but no more than one-half cup of white potato) If the meal includes a grain, it should be whole-grain rich Offer and actively promote half-sized portions for at least 50% of menu items Maximum serving size for a sugary beverage is 16 ounces, and smaller portions are preferred ***Children’s meals:*** **At least 25% of children’s menu items should meet the following criteria:** ≤600 calories/meal ≤35% of calories from total fat/meal ≤10% of calories from saturated fat/meal <0.5 g of TFA/meal ≤35% calories from total sugars/meal ≤770 mg sodium/meal Offer no sugary beverages Include two sources of a vegetable/fruit not including juice; whole grains (>50% of grain ingredients), lean protein (i.e., skinless white meat poultry, fish/seafood, beef, pork, tofu, beans, egg); >2 ounces of meat, 1 egg, 1 ounce of nuts/seeds/dry beans/ peas; and >½ cup 1% or fat-free milk or low-fat dairy.
2014	U.S. National Salt Reduction Initiative Targets for Restaurants [77]	**This initiative set targets** **for 10 restaurant food categories (i.e., hamburgers, chicken, seafood, sandwiches, pizza, potatoes, soup, and bakery products) to meet the following criteria/100 g for items sold by 2014:** ≤1200 mg sodium/serving maximum for all items *Examples:* fries ≤240 mg/100 g; hamburgers ≤330 mg/100 g; and breakfast sandwiches ≤630 mg/100 g
2016	U.S. Expert Panel on Children’s Menu Portions [78]	**A single serving of a children’s menu entree should not exceed the following criteria:** ≤600 calories/meal and ≤300 calories/serving for a la carte items to a main dish ≤100 calories/serving for fried potatoes ≤150 calories/serving for desserts ≤150 calories/serving for soups, appetizers, snacks, and vegetables or salads with added ingredients ≤110 calories/8 ounce (250 mL) serving for non-fat or low-fat milk (children aged 2–4 years) ≤130 calories/8 ounce (250 mL) serving for flavored milk (children aged 4–12 years)

* The contents of this table are summarized from a scoping review of dietary recommendations for restaurant chains conducted in 2018.

**Table 4:** Evaluation of study quality assessed by the Joanna Briggs Institute critical appraisal checklist.

Lead Author, Year	1. Were the Inclusion Criteria for the Sample Clearly Defined?			2. Were the Study Subjects and the Setting Described in Detail?			3. Was the Exposure Measured in a Valid and Reliable Way?			4. Were Objective, Standard Criteria Used to Measure the Condition?			5. Were Compounding Factors Identified?			6. Were Strategies Stated to Manage Confounding Factors?			7. Were the Outcomes Measured in a Valid and Reliable Way?			8. Was the Appropriate Statistical Analysis Used?			Overall Study Quality Score 1 = Weak 2 = Moderate 3 = Strong
*Reviewers’ decision*	1	2	1&2	1	2	1&2	1	2	1&2	1	2	1&2	1	2	1&2	1	2	1&2	1	2	1&2	1	2	1&2	
Ahuja et al., 2015 [79]	Y	Y	Y	Y	Y	Y	Y	Y	Y	Y	Y	Y	N/A	N/A	N/A	N/A	N/A	N/A	Y	Y	Y	Y	Y	Y	3
Astiasarán et al., 2017 [80]	Y	Y	Y	Y	Y	Y	Y	Y	Y	Y	Y	Y	N/A	N/A	N/A	N/A	N/A	N/A	Y	Y	Y	Y	Y	Y	3
Auchincloss et al., 2014 [81]	Y	Y	Y	Y	Y	Y	Y	Y	Y	Y	Y	Y	N	N	N	N	N	N	Y	Y	Y	Y	Y	Y	2
Bauer et al., 2012 [82]	Y	Y	Y	Y	Y	Y	Y	Y	Y	Y	Y	Y	N/A	N/A	N/A	N/A	N/A	N/A	Y	Y	Y	Y	Y	Y	3
Bleich et al., 2015 [83]	Y	Y	Y	Y	Y	Y	Y	Y	Y	Y	Y	Y	Y	Y	Y	Y	Y	Y	Y	Y	Y	Y	Y	Y	3
Bleich et al., 2016 [84]	Y	Y	Y	Y	Y	Y	Y	Y	Y	Y	Y	Y	Y	Y	Y	Y	Y	Y	Y	Y	Y	Y	Y	Y	3
Bleich et al., 2017 [85]	Y	Y	Y	Y	Y	Y	Y	Y	Y	Y	Y	Y	Y	Y	Y	Y	Y	Y	Y	Y	Y	Y	Y	Y	3
Brindal et al., 2008 [86]	Y	Y	Y	Y	Y	Y	Y	Y	Y	Y	Y	Y	N/A	N/A	N/A	N/A	N/A	N/A	Y	Y	Y	N	N	N	2
Bruemmer et al., 2012 [87]	Y	Y	Y	Y	Y	Y	Y	Y	Y	Y	Y	Y	N	N	N	N	N	N	Y	Y	Y	Y	Y	Y	3
Chand et al., 2012 [88]	Y	Y	Y	Y	Y	Y	Y	Y	Y	Y	Y	Y	N/A	N/A	N/A	N/A	N/A	N/A	N	Y	Y	Y	Y	Y	3
Cohen et al., 2017 [78]	Y	Y	Y	Y	Y	Y	Y	Y	Y	Y	Y	Y	N/A	N/A	N/A	N/A	N/A	N/A	Y	Y	Y	Y	Y	Y	3
Deierlein et al., 2015 [89]	Y	Y	Y	Y	Y	Y	Y	Y	Y	Y	Y	Y	N/A	N/A	N/A	N/A		N/A	Y	Y	Y	Y	Y	Y	3
Dunford et al., 2010 [90]	Y	Y	Y	Y	Y	Y	Y	Y	Y	Y	Y	Y	N/A	N/A	N/A	N/A	N/A	N/A	Y	Y	Y	Y	Y	Y	3
Dunford et al., 2012 [91]	Y	Y	Y	Y	Y	Y	Y	Y	Y	Y	Y	Y	N/A	N/A	N/A	N/A	N/A	N/A	Y	Y	Y	Y	Y	Y	3
Eissa et al., 2017 [92]	Y	Y	Y	Y	Y	Y	Y	Y	Y	Y	Y	Y	N/A	N/A	N/A	N/A	N/A	N/A	Y	Y	Y	Y	Y	Y	3
Eyles et al., 2018 [93]	Y	Y	Y	Y	Y	Y	Y	Y	Y	Y	Y	Y	Y	Y	Y	Y	Y	Y	Y	Y	Y	Y	Y	Y	3
Garcia et al., 2014 [94]	Y	Y	Y	Y	Y	Y	Y	Y	Y	Y	Y	Y	N	N	N	N	N	N	Y	Y	Y	Y	Y	Y	3
Garemo and Naimi, 2018 [95]	N	N	N	Y	Y	Y	Y	Y	Y	Y	Y	Y	N/A	N/A	N/A	N/A	N/A	N/A	Y	Y	Y	N	N	N	2
Hearst et al., 2013 [96]	Y	Y	Y	Y	Y	Y	Y	Y	Y	Y	Y	Y	N/A	N/A	N/A	N/A	N/A	N/A	Y	Y	Y	Y	Y	Y	3
Heredia-Blonval et al., 2014 [97]	Y	Y	Y	Y	Y	Y	Y	Y	Y	Y	Y	Y	N/A	N/A	N/A	N/A	N/A	N/A	Y	Y	Y	Y	Y	Y	3
Hobin et al., 2014 [98]	Y	Y	Y	Y	Y	Y	Y	Y	Y	Y	Y	Y	Y	Y	Y	Y	Y	Y	Y	Y	Y	Y	Y	Y	3
Jacobson et al., 2013 [99]	Y	Y	Y	Y	Y	Y	Y	Y	Y	Y	Y	Y	N/A	N/A	N/A	N/A	N/A	N/A	Y	Y	Y	Y	Y	Y	3
Jarlenski et al., 2016 [100]	Y	Y	Y	Y	Y	Y	Y	Y	Y	Y	Y	Y	Y	Y	Y	Y	Y	Y	Y	Y	Y	Y	Y	Y	3
Khan et al., 2018 [101]	Y	Y	Y	N	N	N	N	N	N	N	N	N	N/A	N/A	N/A	N/A	N/A	N/A	N	Y	Y	Y	Y	Y	2
Kirkpatrick et al., 2013 [102]	Y	Y	Y	Y	Y	Y	Y	Y	Y	Y	Y	Y	N/A	N/A	N/A	N/A	N/A	N/A	Y	Y	Y	Y	Y	Y	3
Mazariegos et al., 2016 [103]	Y	Y	Y	Y	Y	Y	Y	Y	Y	Y	Y	Y	N/A	N/A	N/A	N/A	N/A	N/A	Y	Y	Y	Y	Y	Y	3
Moran et al., 2017 [104]	Y	Y	Y	Y	Y	Y	Y	Y	Y	Y	Y	Y	Y	Y	Y	Y	Y	Y	Y	Y	Y	Y	Y	Y	3
O’Donnell et al., 2008 [105]	Y	Y	Y	Y	Y	Y	Y	Y	Y	Y	Y	Y	N/A	N/A	N/A	N/A	Y	Y	Y	Y	Y	Y	Y	Y	3
Prentice et al., 2015 [106]	Y	Y	Y	Y	Y	Y	Y	Y	Y	Y	Y	Y	Y	Y	Y	Y	Y	Y	Y	Y	Y	Y	Y	Y	3
Reeves et al., 2011 [107]	Y	Y	Y	Y	Y	Y	Y	Y	Y	Y	Y	Y	N/A	N/A	N/A	N/A	Y	Y	Y	Y	Y	Y	Y	Y	3
Roberts et al., [108]	Y	Y	Y	Y	Y	Y	Y	Y	Y	Y	Y	Y	Y	Y	Y	Y	Y	Y	Y	Y	Y	Y	Y	Y	3
Rudelt et al., 2014 [109]	Y	Y	Y	Y	Y	Y	Y	Y	Y	Y	Y	Y	N/A	N/A	N/A	N/A	N/A	N/A	Y	Y	Y	Y	Y	Y	3
Schoffman et al., 2016 [110]	Y	Y	Y	Y	Y	Y	Y	Y	Y	Y	Y	Y	Y	Y	Y	Y	Y	Y	Y	Y	Y	Y	Y	Y	3
Scourboutakos and L’Abbé, 2012 [111]	Y	Y	Y	Y	Y	Y	Y	Y	Y	Y	Y	Y	N	N	N	N	N	N	Y	Y	Y	Y	Y	Y	3
Scourboutakos et al., 2013 [112]	Y	Y	Y	Y	Y	Y	Y	Y	Y	Y	Y	Y	N/A	N/A	N/A	N/A	N/A	N/A	Y	Y	Y	Y	Y	Y	3
Scourboutakos et al., 2016 [113]	Y	Y	Y	Y	Y	Y	Y	Y	Y	Y	Y	Y	N/A	N/A	N/A	N/A	N/A	N/A	Y	Y	Y	Y	Y	Y	3
Scourboutakos and L’Abbé, 2013 [114]	Y	Y	Y	Y	Y	Y	Y	Y	Y	Y	Y	Y	N/A	N/A	N/A	N/A	N/A	N/A	Y	Y	Y	Y	Y	Y	3
Scourboutakos et al., 2018 [115]	Y	Y	Y	Y	Y	Y	Y	Y	Y	Y	Y	Y	N/A	N/A	N/A	N/A	N/A	N/A	Y	Y	Y	Y	Y	Y	3
Scourboutakos et al., 2014 [116]	Y	Y	Y	Y	Y	Y	Y	Y	Y	Y	Y	Y	Y	Y	Y	Y	Y	Y	Y	Y	Y	Y	Y	Y	3
Sliwa et al., 2016 [117]	Y	Y	Y	Y	Y	Y	Y	Y	Y	Y	Y	Y	N/A	N/A	N/A	N/A	N/A	N/A	Y	Y	Y	Y	Y	Y	3
Soo et al., 2018 [118]	Y	Y	Y	Y	Y	Y	Y	Y	Y	Y	Y	Y	Y	Y	Y	Y	Y	Y	Y	Y	Y	Y	Y	Y	3
Stender et al., 2006 [119]	Y	Y	Y	Y	Y	Y	Y	Y	Y	Y	Y	Y	N/A	N/A	N/A	N/A	N/A	N/A	Y	Y	Y	Y	Y	Y	3
Uechi, 2018 [120]	Y	Y	Y	Y	Y	Y	Y	Y	Y	Y	Y	Y	Y	Y	Y	Y	Y	Y	Y	Y	Y	Y	Y	Y	3
Urban et al., 2014 [121]	Y	Y	Y	Y	Y	Y	Y	Y	Y	Y	Y	Y	Y	Y	Y	Y	Y	Y	Y	Y	Y	Y	Y	Y	3
Urban et al., 2014 [122]	Y	Y	Y	Y	Y	Y	Y	Y	Y	Y	Y	Y	Y	Y	Y	Y	Y	Y	Y	Y	Y	Y	Y	Y	3
Waterlander et al., 2014 [123]	Y	Y	Y	Y	Y	Y	Y	Y	Y	N	N	N	N	N	N	N	N	N	N	N	N	N	N	N	1
Wellard et al., 2012 [124]	Y	Y	Y	Y	Y	Y	Y	Y	Y	Y	Y	Y	N/A	N/A	N/A	N/A	N/A	N/A	Y	Y	Y	Y	Y	Y	3
Wellard-Cole et al., 2018 [125]	Y	Y	Y	Y	Y	Y	Y	Y	Y	Y	Y	Y	N/A	N/A	N/A	N/A	N/A	N/A	Y	Y	Y	Y	Y	Y	3
Wolfson et al., 2018 [126]	Y	Y	Y	Y	Y	Y	Y	Y	Y	Y	Y	Y	Y	Y	Y	Y	Y	Y	Y	Y	Y	Y	Y	Y	3
Ziauddeen et al., 2015 [127]	Y	Y	Y	Y	Y	Y	Y	Y	Y	Y	Y	Y	N/A	N/A	N/A	N/A	N/A	N/A	Y	Y	Y	Y	Y	Y	3

Y = yes; N = no; N/A = not applicable.

**Table 5:** Studies included in the systematic review summarized by country, study design, methods, data collection period, outcomes measured, and chains examined, 2000–2018.

Lead Author, Year	Country	Study Design	Methods and Data Sources	Data Collection Period	Outcomes Measured #: Type	Chains Examined #: Type
Ahuja et al., 2015 [79]	USA	Cross-sectional	Menu info from restaurant websites, meal samples, lab analyses	2010–2013	2: sodium (mg), sodium density (mg/100 g)	4 QSR chains
Astiasarán et al., 2017 [80]	Spain	Cross-sectional	Lab analysis of fries using gas chromatography	2017	4: energy (kcal), energy density (kcal/100 g), fat (g), TFA (g/100 g fat)	5 QSR chains
Auchincloss et al., 2014 [81]	USA	Cross-sectional	Menu info from restaurant websites	2011	4: energy (kcal), saturated fat (g), sodium (mg), sodium density (mg/1000 kcal)	21 QSR, FCR and FSR chains
Bauer et al., 2012 [82]	USA	Longitudinal	Menu info from restaurant websites analyzed by University of Minnesota Nutrient Database	2006–2010	3: energy (kcal), saturated fat (g), sodium (mg)	8 QSR chains
Bleich et al., 2015 [83]	USA	Longitudinal	MenuStat Database	2012–2013	1: energy (kcal)	66 QSR, FCR and FSR chains
Bleich et al., 2016 [84]	USA	Longitudinal	MenuStat Database	2012–2014	1: energy (kcal)	66 QSR, FCR and FSR chains
Bleich et al., 2017 [85]	USA	Longitudinal	MenuStat Database	2008 and 2012–2015	1: energy (kcal)	44 QSR, FCR and FSR chains
Brindal et al., 2008 [86]	Australia	Cross-sectional	Menu info from restaurant websites, onsite visits, phone calls	2005	3: energy (kcal), fat (g), saturated fat (g)	6 QSR chains
Bruemmer et al., 2012 [87]	USA	Cross-sectional	Menu info from restaurant onsite visits and audits	2009–2010	3: energy (kcal), saturated fat (g), sodium (mg)	37 QSR, FCR and FSR chains
Chand et al., 2012 [88]	New Zealand	Cross-sectional	Menu info from restaurant websites, onsite visits, phone calls	2010–2011	5: energy (kJ), fat (g), saturated fat (g), sugar (g), sodium (mg)	12 QSR chains
Cohen et al., 2016 [78]	USA	Cross-sectional	Menustat Database and Delphi method to survey nutrition experts (*n* = 15) about ideal portion size for children’s menu items	2012–2016	2: energy (kcal), portion size	200 QSR, FCR and FSR and non-chain restaurants
Deierlein et al., 2015 [89]	USA	Cross-sectional	Menu info from restaurant websites	2010 and 2014	6: energy (kcal), energy from fat (%), fat (g), saturated fat (g), energy from saturated fat (%), sodium (mg)	29 QSR, FCR and FSR chains
Dunford et al., 2010 [90]	Australia	Cross-sectional	Survey of menu info from restaurant websites	2009	5: energy (kcal), fat (g), saturated fat (g), sugar (g), sodium (mg)	9 QSR chains
Dunford et al., 2012 [91]	Australia, Canada, France, New Zealand, UK, USA	Cross-sectional	Survey of menu info from restaurant websites	2010	2: salt (mg), sodium density (mg/100 g)	6 QSR chains
Eissa et al., 2017 [92]	USA	Cross-sectional	MenStat Database	2012–2014	5: fat (g), saturated fat (g), TFA (g), sugar (g), portion size (g)	42 QSR and FSR chains
Eyles et al., 2018 [93]	New Zealand	Cross-sectional	Annual surveys of menu info from restaurant websites	2012–2016	4: energy (kJ), energy density (kJ/100 g), sodium (mg), portion size (g)	10 QSR chins
Garcia et al., 2014 [94]	Australia	Cross-sectional	Survey of menu info from restaurant websites	2009–2012	2: sodium (mg), sodium density (mg/100 g and mg/serving)	6 QSR chains
Garemo and Naimi, 2018 [95]	UAE	Cross-sectional	Children’s menus collected and analyzed combined with onsite visits to question restaurant staff	2016	3: energy (kcal), fat (g), sugar (g)	58 restaurants
Hearst et al., 2013 [96]	USA	Sequential cross-sectional	Menu info from restaurant websites analyzed by University of Minnesota Nutrient Database	2001–2002, 2003–2004, 2005–2006, 2007–2008, 2009–2010	3: energy (kcal), sodium (g), saturated fat (g)	8 QSR chains
Heredia-Blonval et al., 2014 [97]	Costa Rica	Cross-sectional	Menu info from restaurant websites	2013	3: energy (kcal), salt (mg), sodium density (mg/100 g and mg/serving)	7 QSR chains
Hobin et al., 2014 [98]	Australia, Canada, New Zealand, UK, USA	Cross-sectional	Children’s menu info from restaurant websites or phone calls	2012	5: energy (kcal), fat (g), saturated fat (g), sodium (mg), serving size (g)	4 QSR chains
Jacobson et al., 2013 [99]	USA	Longitudinal	Menu info from restaurant websites	2005–2011	1: sodium (g)	16 QSR and FCR chains
Jarlenski et al., 2016 [100]	USA	Longitudinal	MenuStat Database	2012–2014	4: energy (kcal), fat (g), saturated fat (g), sugar (g)	37 QSR and FCR chains
Khan et al., 2018 [101]	Australia, Egypt, India, USA	Cross-sectional	Menu info from restaurant websites or print materials	2015	2: energy (kcal), sodium density (g/100 g)	3 QSR chains
Kirkpatrick et al., 2013 [102]	USA	Cross-sectional	Children’s menu info from restaurant websites	2008–2009	5: energy (kcal), energy from fat (%), energy from added sugars (%), saturated fat (g), sodium (g)	5 QSR chains
Mazariegos et al., 2016 [103]	Guatemala	Cross-sectional	Children’s menu info from restaurant websites, onsite visits or phone calls	2016	6: energy (kcal), sodium (mg), sugar (g), TFA (g), saturated fat (%), energy from fat (%)	6 QSR chains
Moran et al., 2017 [104]	USA	Cross-sectional	Children’s menu items from MenuStat Database	2012–2015	3: energy (kcal), sodium (mg), saturated fat (g)	45 QSR, FCR and FSR chains
O’Donnell et al., 2008 [105]	USA	Cross-sectional	Menu items from restaurant websites and phone calls	2007	6: energy (kcal), fat (g), energy from fat (%), saturated fat (g), sugars (g), sodium (mg)	10 QSR chains
Prentice et al., 2015 [106]	New Zealand	Cross-sectional	Menu items from restaurant websites	2008–2009	1: sodium (mg)	8 QSR chains
Reeves et al., 2011 [107]	England	Cross-sectional	Menu items from restaurant websites	2009	4: energy (kcal), fat (g), sodium (mg), portion size (g)	7 QSR chains and 15 non-chain FSR
Roberts et al., [108]	Brazil, China, Finland, Ghana, India, USA	Cross-sectional	Menu items from restaurant websites, onsite visits, and lab analysis of selected items using bomb calorimetry	2014 and 2017	2: energy (kcal), energy density (kcal/g)	111 QSR and FSR chains
Rudelt et al., 2014 [109]	USA	Cross-sectional	Menu info from restaurant websites analyzed by University of Minnesota Nutrient Database	2000 and 2009/2010	1: sodium (mg)	8 QSR chains
Schoffman et al., 2016 [110]	USA	Cross-sectional	MenuStat Database	2014	1: energy (kcal)	62 QSR and FCR chains
Scourboutakos and L’Abbé, 2012 [111]	Canada	Cross-sectional	Menu items from restaurant websites	2010	3: energy (kcal), energy density (% kcal/100 g food), portion size (g)	85 QSR and FSR chains
Scourboutakos et al., 2013 [112]	Canada	Cross-sectional	Menu items from restaurant websites	2910–2011	5: energy (kcal), fat (g), saturated fat (g), TFA (g), sodium (mg)	19 FSR chains
Scourboutakos et al., 2016 [113]	Canada	Cross-sectional	Menu items from restaurant websites	2010	2: total sugar (g), added sugar (g)	17 QSR and FSR chains
Scourboutakos and L’Abbé, 2013 [114]	Canada	Cross-sectional	Canadian MENU-Food Label Information Program (FLIP) Database of nutrition info of restaurant menu items	2010	1: sodium (mg)	85 QSR and FSR chains
Scourboutakos et al., 2018 [115]	Canada	Longitudinal	Menu items from restaurant websites	2010–2016	1: sodium (mg)	12 QSR, FCR and FSR chains
Scourboutakos et al., 2014 [116]	Canada	Longitudinal	Menu items from restaurant websites	2010–2013	4: energy (kcal), sodium (mg), sodium density (mg/100 g), portion size (g)	61 QSR, FCR and FSR chains
Sliwa et al., 2016 [117]	USA	Cross-sectional	Children’s menu info from restaurant websites	2014	5: energy (kcal), fat (g), saturated fat (g), sodium (mg), portion size (g)	20 QSR, FCR and FSR chains
Soo et al., 2018 [118]	USA	Cross-sectional	Menu info from restaurant signs and menu boards	2010 and 2013	5: energy (kcal), saturated fat (g), sugar (g), sodium (mg), portion size (g)	4 QSR chains
Stender et al., 2006 [119]	Austria, Czech Republic, Denmark, England, Hungary Finland, France Germany, Italy Netherlands, Norway Peru, Poland Portugal, Russia Scotland, Spain South Africa, Sweden, USA	Cross-sectional	Lab analysis of two items (i.e., chicken and fries) at each restaurant chain using gas chromatography	2004 and 2005	1: TFA (g)	2 QSR chains
Uechi, 2018 [120]	Japan	Cross-sectional	Children’s menu info from restaurant websites	2017	4: energy (kJ), sugar (g), fat (g), sodium (mg)	20 restaurant chains
Urban et al., 2014 [121]	USA	Longitudinal	Children’s menu info from restaurant websites	2000–2013	4: energy (kcal), sodium (mg), saturated fat (g), TFA (g)	3 QSR chains
Urban et al., 2014 [122]	USA	Longitudinal	Children’s menu info from restaurant websites	2000–2013	3: sodium density (mg/1000 kcal), saturated fat (g/1000 kcal), TFA (g/1000 kcal)	3 QSR chains
Waterlander et al., 2014 [123]	New Zealand	Cross-sectional	Children’s menu info from restaurant websites	2014	4: energy (kcal), saturated fat (g), sugar (g), sodium (mg)	4 QSR chains
Wellard et al., 2012 [124]	Australia	Cross-sectional	Children’s menu info from restaurant websites	2010	4: energy (kJ), saturated fat (g), sugar (g), sodium (mg)	6 QSR chains
Wellard-Cole et al., 2018 [125]	Australia	Cross-sectional	Children’s menu info from restaurant websites	2009 and 2015	2: energy (kJ), energy density (kJ/100 g and kJ/serving)	5 QSR chains
Wolfson et al., 2018 [126]	USA	Cross-sectional	MenuStat Database	2012 and 2016	1: sodium (mg)	66 QSR, FCR and FSR chains
Ziauddeen et al., 2015 [127]	Australia, Canada, China, Germany, Japan, Netherlands, New Zealand, UAE, UK, USA	Cross-sectional	Menu info from restaurant websites	2012	3: energy (kcal), fat (g), saturated fat (g)	5 QSR chains

**Abbreviations:** kilocalories (kcal); kilojoules (kJ); milligrams (mg); fast-casual restaurants (FCR); full-service restaurants (FSR); grams (g); milligrams (mg); quick-service restaurants (QSR); trans fatty acids (TFA); United Arab Emirates (UAE); United Kingdom (UK); United States of America (USA).

## Supplementary Materials

The following are available online at https://www.mdpi.com/1660-4601/16/15/2732/s1, Table S1: Detailed search strategy used for the systematic review, Table S2: Published studies (*n* = 50) of transnational restaurant chains to reformulate products and standardize portions to meet healthy dietary guidelines, 2000–2018, Table S3: Published studies (*n* = 50) of transnational restaurant chains to reformulate products and standardize portions to meet healthy dietary guidelines by geographic region, 2000–2018.
