# Supplementary material for: Progress Evaluation for Transnational Restaurant Chains to Reformulate Products and Standardize Portions to Meet Healthy Dietary Guidelines and Reduce Obesity and Non-Communicable Disease Risks, 2000–2018: A Scoping and Systematic Review to Inform Policy
Source: Int J Environ Res Public Health. 2019 Jul 31;16(15):2732. doi: 10.3390/ijerph16152732 (PMC6695776; doi:10.3390/ijerph16152732)
Supplement: Supplementary file 1 [file ijerph-16-02732-s001.zip › IJERPH-527136-suppl xml.docx]

**Supplemental Table 1.** Detailed search strategy used for the systematic review. The five electronic databases searched on 18 October 2018 were CINAHL, Food Science Technology Abstracts, Mintel, PubMed and Web of Science. The detailed search terms are listed in the table below for each database.

| **Database** | **Search Terms** |
| --- | --- |
| **CINAHL: 23 records identified** | TI Restaurant OR TI Restaurants OR TI Fast food OR TI Fast foods OR TI Takeaway OR TI Takeout  AND Reformulation OR Reformulations OR Change OR Portion OR Portions OR serving size OR serving portion OR Standardized serving OR Standardized portion OR Serving OR servings  AND dietary guidelines OR dietary guideline OR diet OR ( recommendations or guidelines ) OR nutrition guidelines OR nutritional standards OR nutrition policy OR food policy OR ( monitoring and evaluation )  AND energy OR calories OR calorie OR caloric OR kilojoule OR sodium OR salt OR sugar OR saturated fat OR trans fat OR nutrients |
| **Food Science and Technology Abstract: 36 records identified** | (TI Restaurant OR TI Restaurants OR TI Fast food OR TI Fast foods OR TI Takeaway OR TI Takeout ) AND ( Reformulation OR Reformulations OR Change OR Portion OR Portions OR serving size OR serving portion OR Standardized serving OR Standardized portion OR Serving OR servings ) AND ( dietary guidelines OR dietary guideline OR diet OR ( recommendations or guidelines ) OR nutrition guidelines OR nutritional standards OR nutrition policy OR food policy OR ( monitoring and evaluation ) ) AND ( energy OR calories OR calorie OR caloric OR kilojoule OR sodium OR salt OR sugar OR saturated fat OR trans fat OR nutrients ) |
| **MINTEL: 34 records identified** | Restaurant OR “fast food” OR “takeaway” OR “takeout” AND “food reformulation” OR change OR portion OR serving AND guideline OR policy OR regulation OR standard AND energy OR calories or salt OR sugar OR fat |
| **PUBMED: 63 records identified** | (((((restaurant[All Fields] OR restaurant'[All Fields] OR restaurant's[All Fields] OR restaurants [All Fields] OR restaurante[All Fields] OR restaurantes[All Fields] OR restauranteur[All Fields] OR restauranteurs[All Fields] OR restaurantrelateret[All Fields] OR restaurants[All Fields] OR restaurants'[All Fields] OR restaurantswere[All Fields]) AND Title/Abstract[All Fields] OR "Fast food"[All Fields]) AND Title/Abstract[All Fields] OR "Fast foods"[Title/Abstract]) AND (((((((((((((Reformulation[All Fields] OR Reformulations[All Fields]) OR (change[All Fields] OR Portion[All Fields]) OR Portions[All Fields]) OR Serving[All Fields]) OR "Serving size"[All Fields]) OR "Serving sizes"[All Fields]) OR "serving portion"[All Fields]) OR "serving portions"[All Fields]) OR (standardized[All Fields] AND serving[All Fields])) OR (standardized[All Fields] AND servings[All Fields])) OR "standardized portion"[All Fields]) OR "standardized portions"[All Fields])) AND ((((((((((((("dietary guideline"[All Fields] OR "dietary guidelines"[All Fields]) OR ("diet"[MeSH Terms] OR "diet"[All Fields])) OR ("diet"[MeSH Terms] OR "diet"[All Fields] OR "diets"[All Fields])) OR "dietary recommendation"[All Fields]) OR "dietary recommendations"[All Fields]) OR "nutrition standard"[All Fields]) OR "nutrition standards"[All Fields]) OR "nutrition policy"[All Fields]) OR "nutrition policies"[All Fields]) OR "food policy"[All Fields]) OR "food policies"[All Fields]) OR ("monitoring, physiologic"[MeSH Terms] OR ("monitoring"[All Fields] OR "monitor"[All Fields])) OR monitoring[All Fields])) AND ((((((((((((((((("Energy (Oxf)"[Journal] OR "energy"[All Fields]) OR calorie[All Fields]) OR calories[All Fields]) OR caloric[All Fields]) OR (kilojoule[All Fields] OR kilojoules[All Fields] OR kilojoules'[All Fields])) OR (kilocalorie[All Fields] OR kilocalories[All Fields] OR kilocaloriesdagger[All Fields])) OR ("sodium, dietary"[MeSH Terms] OR ("sodium"[All Fields] AND "dietary"[All Fields]) OR "dietary sodium"[All Fields] OR "sodium"[All Fields] OR "sodium"[MeSH Terms]))OR ("sodium"[All Fields] AND "chloride"[All Fields]) OR "salt"[All Fields])) OR fat[All Fields]) OR ("fats"[MeSH Terms] OR "fats"[All Fields])) OR "trans fat"[All Fields]) OR "trans fats"[All Fields]) OR "saturated fat"[All Fields]) OR "saturated fats"[All Fields]) OR (sugar[All Fields] OR sugar'[All Fields] OR sugar''[All Fields] OR sugar'n'[All Fields] OR sugar's[All Fields] OR ("food"[MeSH Terms] OR "food"[All Fields] OR "nutrient"[All Fields])) OR ("food"[MeSH Terms] OR "food"[All Fields] OR "nutrients"[All Fields])) |
| **WEB OF SCIENCE: 23 records identified** | TI=(restaurant OR "fast food" OR restaurants OR "fast foods" OR “takeaway” OR “takeout”) AND  TS=(reformulation OR reformulations OR change)  AND TS=(portion OR portions OR serving OR servings OR "serving size" OR "serving sizes" OR "serving portion" OR "serving portions" OR "standardized serving" OR "standardized servings" OR "standardized portion" OR "standardized portions")  AND TS=("dietary guideline" OR "dietary guidelines" OR diet OR diets OR "dietary recommendation" OR "nutrition standard" OR "nutrition standards" OR "nutrition policy" OR "nutrition policies" OR "food policy" OR "food policies" OR "monitoring" OR "monitor")  AND TS=(energy OR calorie OR calories OR caloric OR kilojoule OR kilocalorie OR kilocalories OR sodium OR salt OR fat OR fats OR "trans fat" OR "trans fats" OR "saturated fat" OR "saturated fats" OR "sugar" OR "nutrient" OR "nutrients") |

**Supplemental Table 2.** Published studies included in the systematic review of transnational restaurant chains to reformulate products and standardize portions to meet healthy dietary guidelines, 2000-2018. *The citations below [78-127] correspond to the text in the manuscript.*

| **Lead author, year** | **Study objective**  **Study location**  *continent*: city, state or country | **Data collection period**  *weeks, months or years*  **Study design** | **Outcomes measured**  **Assessment/evidence sources**  **Dietary guidelines/criteria** | **Restaurant**  **chains examined** | **Results** |
| --- | --- | --- | --- | --- | --- |
| Ahuja et al. 2015 [^[[1]](#endnote-1)^]  Text citation [79] | Monitor sodium content in commercially processed QSR foods.  *North America:*  USA | 2010–2013  Descriptive  cross-sectional | **Outcomes (n=2)**  Sodium (mg)  Sodium density  (mg/100 g)  **Assessment/evidence**  Annual tracking of restaurant foods based on information from restaurants, nationwide sampling, and laboratory analyses.  **Guidelines/criteria**  Sodium compared to What We Eat in America 2007-2008; Dietary Guidelines for Americans (DGA) 2010; Food and Drug Administration (FDA) sodium targets (mg); and the Healthy Eating Index (HEI) 2010. | **4 QSR chains**  McDonald’s Burger King Domino’s  Pizza Hut | A majority (88%; 29 of 33) of food samples at QSR chains exceeded the FDA’s targets for sodium. |
| Astiasarán et al. 2017 [^[[2]](#endnote-2)^]  Text citation [80] | Examine the *trans* fatty acid (TFA) content of French fries at QSR chains.  *Europe:* Pamplona, Navarra, Spain | 2017  Descriptive  cross-sectional | **Outcomes (n=4)**  Energy (kcal)  Energy density  (kcal/100 g)  Fat (g)  Trans fats (TFA)  (g/100 g fat)  **Assessment/evidence**  French fries (n=15 samples) purchased from QSR chains tested using gas chromatography  Results analyzed using Stata v12 software  **Guidelines/criteria**  TFA < 2% total energy | **5 QSR chains**  Not specified | The TFA content for the fries ranged from 0.49% to 0.89%, which was lower than the 2% of total energy set by European countries as the maximum legal content of TFA in fats and contained < 0.5 g/serving. The TFA content of fries were below the target level at five QSR chains in Spain. |
| Auchincloss et al. 2014 [^[[3]](#endnote-3)^]  Text alignment [81] | Assess the nutritional value of meals at QSR, FCR and FSR chains.  *North America:*  Philadelphia, Pennsylvania, USA | Mar and May 2011  Descriptive  cross-sectional | **Outcomes (n=4)**  Energy (kcal)  Saturated fat (g)  Sodium (mg)  Sodium density (mg/1,000 kcal)  **Assessment/evidence**  Data collected from restaurant websites and print menus and outcomes calculated for each menu item. Results analyzed using SAS v 9.2.  **Guidelines/criteria**  DGA 2010 and % Dietary Reference Value (DRV) for a 2000-calorie diet for adults and 1400 calories for children | **21 QSR, FCR and FSR chains**  Denny’s  Friendly’s IHOP  Pizza Hut Applebee’s Neighborhood Grill & Bar Bertucci’s Italian Restaurant California Pizza Kitchen Champs Americana Chili’s Grill & Bar Famous Dave’s Legendary Pit Bar-B-Que Hard Rock Café Houlihan’s Longhorn Steakhouse  Olive Garden  Red Lobster  Ruby Tuesday | Energy content for *à la carte* entrees and appetizers averaged 800 calories, which did not meet healthier criteria for calories 47% of the time. About 30% of *à la carte* entrees exceeded the % DRV for saturated fat and sodium. |
| Bauer et al. 2012 [^[[4]](#endnote-4)^]  Text alignment [82] | Examine changes in the energy content of lunch/dinner menu offerings at QSR chains between 1997/1998 and 2009/2010  *North America:*  USA | 2006–2010  Descriptive  longitudinal | **Outcomes (n=3)**  Energy (kcal)  Saturated fat (g)  Sodium (mg)  **Assessment/evidence**  Data collected from websites and analyzed using the University of Minnesota  Nutrition Coordinating Center’s Food and Nutrient Database.  **Guidelines/criteria**  Not reported | **8 QSR chains**  McDonald’s  Burger King  Wendy’s  Taco Bell  Kentucky Fried Chicken (KFC)  Arby’s  Jack in the Box  Dairy Queen | Median energy content for the general menu, entrée and beverage items did not differ between 2006 and 2010. Energy content of side dishes decreased, but increased for condiments and desserts from 2006-2010. |
| Bleich et al. 2015 [^[[5]](#endnote-5)^]  Text alignment [83] | Describe trends in calories available at U.S. chain restaurants.  *North America:*  USA | 2012–2013  Descriptive longitudinal | **Outcomes (n=1)**  Energy (kcal)  **Assessment/evidence**  Data collected from MenuStat Database 2012 and 2013 (n=19,417 items) that contained menu items reported by restaurants on their websites. Generalized linear models used to calculate mean change in calories from 2012 to 2013, among items on the menu in both years; and difference in mean calories, comparing newly introduced items to those on the menu in 2012 only, overall and between core versus non-core items.  Data analyzed in 2014.  **Guidelines/criteria**  Not reported | **66** QSR, FCR and FSR **chains**  Not specified | Menu items offered in 2012 or 2013 did not significantly reduce calories. Newly introduced items in 2013 had lower calories (-56) than similar 2012 items. Calorie declines were among new main-course items (-10%, -67 calories), new beverages (-8%, -26 calories) and children’s menus (-20%, -46 calories). |
| Bleich et al. 2016 [^[[6]](#endnote-6)^]  Text alignment [84] | Examine trends in calories available in QSR, FCR and FSR chains over two years.  *North America:*  USA | 2012–2014  Descriptive longitudinal | **Outcomes (n=1)**  Energy (kcal)  **Assessment/evidence**  Data collected from the MenuStat Database over three years 2012-2013 and 2014 (n=23,066 items) reported by restaurants on their websites. Statistical significance was set at p<0.05.  **Guidelines/criteria**  Not reported | **66 QSR, FCR and FSR chains**  Not specified | Calories in newly introduced menu items declined by 71 (15%) from 2012 to 2013 (p=0.001) and by 69 (or 14%) from 2012 to 2014 (p=0.03). Declines were in new main course items (85 fewer calories in 2013 and 55 fewer calories in 2014; p=0.01). Average calories in newly introduced menu items are declining but are higher than items common to the menu in all 3 years. No differences were found in mean calories among items on menus in 2012, 2013, or 2014. |
| Bleich et al. 2017 [^[[7]](#endnote-7)^]  Text alignment [85] | Describe trends in calories available at large U.S. chain restaurants over seven years.  *North America:*  USA | 2008 and 2012–2015  Descriptive  longitudinal | **Outcomes (n=1)**  Energy (kcal)  **Assessment/evidence**  MenuStat Database 2012-2015 that contained menu items reported by restaurants on their websites. Percentage of menu items available from 2012 to 2015 and in 2008 were calculated. Analysis examined mean within-item change in calories from 2008 to 2015, among items on the menu in all years; and the difference in mean per-item calories, comparing menu items newly introduced in 2012, 2013, 2014 and 2015 to those items on the menu in 2008 only.  **Guidelines/criteria**  Not reported | **44 QSR, FCR and FSR chains**  Not specified | Items common to the menu in all years had an overall decline on calories from 327 kcal in 2008 to 318 kcal in 2015. No observed differences in mean calories among newly introduced menu items in 2012, 2013, 2014, and 2015 relative to items only on the menu in 2008. US national menu labeling mandate may have influenced restaurants to lower the average calories for menu items. |
| Brindal et al. 2008 [^[[8]](#endnote-8)^]  Text alignment [86] | Compare the macronutrient  content of QSR meals and healthier choices from restaurant chains.  *Oceania:* Australia | Oct 2005  Descriptive  cross-sectional | **Outcomes (n=3)**  Energy (kJ)  Fat (g)  Saturated fat (g)  **Assessment/evidence**  Nutrition information for both types of meals were obtained from restaurant websites, follow-up phone calls and visits; and converted into a % Daily Allowance.  **Guidelines/criteria**  8400 kJ/day for average adult. Fat or saturated fat guidelines or targets not reported. | **6 QSR chains**  Domino’s  Hungry Jack’s  KFC  McDonald’s  Red Rooster  Subway | Average meal provided nearly half (47.5%) of the total energy (kJ) and dietary fat (47.1% and 93.5%), respectively, compared to the recommended daily targets. |
| Bruemmer et al. 2012 [^[[9]](#endnote-9)^]  Text alignment [87] | Evaluate selected nutrient content of entrées 6- and 18-months after implementation of a US county restaurant menu labeling law  *North America:* King County of Washington State,  USA | May–Jun 2009 (pre)  May–Jun 2010 (post)  Descriptive  cross-sectional | **Outcomes (n=3)**  **Energy (kcal)**  **Saturated fat (g)**  **Sodium (mg)**  **Assessment/evidence**  Audited the content of menu items at one restaurant per chain at 6 and 18 months after menu labeling legislation enacted, compared with one-third of the recommended daily nutrient intake for adults.  **Guidelines/criteria**  DGA 2005 | **37 QSR, FCR and FSR chains**  Not specified | Energy content was lower at FSR chains (-7%, -73 calories) and LSR chains (-3%, -19 calories) from 2009 to 2010. All chains exceeded the DGA 2005 recommendations for calories (56%), saturated fat (77%), and sodium (89%). |
| Chand et al. 2012 [^[[10]](#endnote-10)^]  Text alignment [88] | Assess availability of healthier options at QSR chains.  *Oceania:*  New Zealand | Dec 2010 –  Jan 2011  Descriptive  cross-sectional | **Outcomes (n=5)**  Energy (kJ)  Fat (g)  Saturated fat (g)  Sugar (g)  Sodium (mg)  **Assessment/evidence**  Onsite visits combined with phone calls and website searches to identify products (n=1126) sold at QSR chains.  Nutrient composition of healthier versus regular meals per serving.    **Guidelines/criteria**  Not reported | **12 QSR chains**  Burger Fuel  Burger King  Burger Wisconsin  Domino’s  Hell Pizza  KFC  McDonald’s  Muffin Break  Pizza Hut  Starbucks  Subway  Tank Juice | One-fifth (21%; n=234/1126) of products met healthier dietary guidelines, defined by QSR chain as ‘healthier’, ‘lite’ or having smaller portion size.  Meal options were high in sugar or sodium per serving. Mean sugar content of beverages was 56 g (11 teaspoons/serving); and sodium content of burgers was 1095 mg/serving, and pasta dishes were 1172 mg sodium/serving. |
| Cohen et al. 2016 [^[[11]](#endnote-11)^]  Text alignment [78] | Examine calories and portion sizes of children’s meals.  *North America:* USA | 2012-2016  Descriptive  cross-sectional | **Outcomes (n=2)**  Energy (kcal)  Portion size  **Assessment/evidence**  Examined calories and portions of  items sold restaurant chains using the MenuStat Database 2014, then a Delphi Method to poll national childhood nutrition experts (n=15) to assess the ideal portion sizes for various food categories.  **Guidelines/criteria**  ≤ 600 calories/meal | **200 restaurant chains**  Not specified | Only 54% (108 of 200 restaurants) in MenuStat Database publicly disclosed nutritional data for children’s menu items.  Actual portion size/calorie content recorded for 200 U.S. restaurant chains exceeded the recommended amounts for children’s *à la carte* items (≤ 300 kcal/serving), side dishes and dessert (≤ 150 calories/serving), and entrees (≤ 600 kcal/serving), with the exception of fruit and vegetables, which were 46% and 69% of the recommended calorie content, respectively.  FSR chains were more likely to serve children’s menu items exceeding 600 calories/serving. Chains that served highest calorie entrée was two mini Angus cheeseburgers (1170 kcal). |
| Deierlein et al. 2015 [^[[12]](#endnote-12)^]  Text alignment [89] | Determine changes in the nutritional content of children’s menu items at chain restaurants over four years.  *North America:* USA | Jun-Jul  2010 and 2014  Descriptive  cross-sectional | **Outcomes (n=6)**  Energy (kcal)  Energy from fat (%)  Fat (g)  Saturated fat (g)  Energy from saturated fat (%)  Sodium (mg)  **Assessment/evidence**  Sample consisted of chains ranked in top 50 in 2009.  Nutritional information was accessed in 2010 and 2014 from restaurant chains’ websites. Differences in means of nutrient content or percent of dishes with fruits or vegetables between 2010 and 2014 at QSR and FSR chains evaluated by t-tests and chi-square tests  (p < 0.05).  **Guidelines/criteria**  DGA 2010 target for sodium < 2300 mg/day | **29 QSR, FCR and FSR chains**  Not specified | Nutrient content of main dishes for children did not change significantly between 2010 and 2014.  A majority of children’s menu items, especially entrees, offered high amounts of calories, fat, saturated fat, and sodium compared to the DGA 2010 targets. One-third of main dishes at QSR chains and half of main dishes at FSR chains exceeded the 2010 DGA target for sodium, fat, and saturated fat in 2014.  Improvements in nutrient content were observed for side dishes. At FSR chains, added side dishes contained over 50% less calories, fat, saturated fat, and sodium, and were more likely to contain fruits/vegetables compared to removed sides (p < 0.05 for all comparisons). Added side dishes at QSR chains contained less saturated fat (p < 0.05). |
| Dunford et al. 2010 [^[[13]](#endnote-13)^]  Text alignment [90] | Examine the nutrient content of QSR menu items compared to healthy dietary guidelines.  *Oceania:* Australia | Jun 2009  Descriptive  cross-sectional | **Outcomes (n=5)**  Energy (kcal)  Fat (g)  Saturated fat (g)  Sugar (g)  Sodium (mg)  **Assessment/evidence**  Mean nutrient levels  were compared between product categories and with recommended healthy nutrient criteria.    Data were collected by a survey of menu items from websites and nutrient content was calculated for products/serving and per 100 g.  **Guidelines/criteria**  Nutrient criteria set by the UK’s Food Standards Agency (FSA) and products were classified accordingly as ‘high’, ‘moderate’ or ‘low’ | **9 QSR chains**  McDonald’s  Hungry Jack’s Oporto  KFC  Red Rooster  Pizza Hut  Domino’s  Eagle Boys  Subway | Majority of products did not meet healthy criteria. Breakfast items had the highest mean sugar content (7.8 g/100 g) and saturated fat (5.5 g/100 g), and chicken items had the highest total fat (13.2 g/100 g) and sodium content (586 mg/100 g), and sides had the highest mean energy content (1087 kJ/100 g).  Variation in the nutrient content of comparable products across the chains implicated the potential for product reformulation across all product categories that could have substantial impact on reducing poor dietary quality if all firms adhered to common nutrient targets. |
| Dunford et al. 2012 [^[[14]](#endnote-14)^]  Text alignment [91] | Examine and compare the sodium content of foods sold at QSR chains in six countries.  *Europe:* France and United Kingdom  *North America:* Canada and USA  *Oceania:* Australia and  New Zealand | Apr 2010  Descriptive  cross-sectional | **Outcomes (n=2)**  Salt (mg)  Sodium density  (mg/100 g)  **Assessment/evidence**  Data obtained from chain websites in each country for seven food categories (i.e., savory breakfast items, burgers, chicken products, pizza, salads, sandwiches and fries). Mean levels and ranges for salt for each food category and separately for each chain were calculated across six countries.  **Guidelines/criteria**  Not reported | **6 QSR chains**  Burger King (Hungry Jack’s)  Domino’s Pizza  KFC  McDonald’s  Pizza Hut  Subway | The salt content varied substantially by food category and QSR chain in countries. Salads contained 0.5 g salt/100 g, whereas the chicken products contained 1.6 g salt/100 g. We also saw variability between countries: chicken products from the UK contained 1.1 g of salt per 100 g, whereas chicken products from the US contained 1.8 g. Furthermore, the mean salt content of food categories varied between companies and between the same products in different countries  (e.g., McDonald’s Chicken McNuggets contained 0.6 g of salt per 100 g in the UK, but 1.6 g of salt per 100 g in the US). |
| Eissa et al. 2017 [^[[15]](#endnote-15)^] Text alignment [92] | Examine the nutrition content of children's menu items at QSRs and FSRs compared to DGA 2015-2020.  *North America:* USA | 2012-2014  Descriptive  Cross-sectional | **Outcomes (n=5)**  Fat (g)  Saturated fat (g)  TFA (g)  Sugar (g)  Portion size (g)  **Assessment/evidence**  Using the MenuStat Database 2014, 10 food items on QSR and FSR children's menus were selected. Data from each restaurant category were aggregated, and overall average of the nutritional content of each individual food item was calculated and compared between the two chain categories.  Data were collected from restaurant websites.  **Guidelines/criteria**  Daily recommended calories based on the American Academy of Pediatrics target of  1200-1600 kcal/day and DGA 2015-2020 of  1550-1650 kcal/day | **42 QSR and FSR chains**  Not specified | Most menu items at FSR and QSR chains did not meet DGA 2015-2020 targets. Average for calories, fat, and added sugar of most items on the children’s menus were lower at QSR chains compared to FSR chains. Most food items on children's menus at FSRs, and to a lesser extent at QSRs, exceeded the national recommended calories and fat content per meal. The difference between nutrient content means of FSR and QSR menu items were statistically significant at p<0.05. |
| Eyles et al. 2018 [^[[16]](#endnote-16)^]  Text alignment [93] | Examine nutrient content and serving/portion size changes of menu items sold annually for four years at QSR chains.  *Oceania:* New Zealand | Feb and Mar 2012-2016  Descriptive sequenced, annual cross-sectional | **Outcomes (n=4)**  Energy (kJ)  Energy density  (kJ per 100 g)  Sodium (mg/serving)  Portion size (g)  **Assessment/evidence**  Serving/portion size and nutrient data were collected in annual cross-sectional surveys of all products sold at 10 QSR chains over 4 years from restaurant websites.  **Guidelines/criteria**  Not reported | **10 QSR chains**  Burger King  Domino’s  Hell Pizza  KFC  McDonald’s Corporation  Muffin Break  Pizza Hut  St Pierre's Sushi  Subway  Tank Juice | Moderate to large increases in the mean serving size, energy density, energy/serving, and sodium/serving, except for sodium density, were observed across all menu items examined between 2012 and 2016. |
| Garcia et al. 2014 [^[[17]](#endnote-17)^]  Text alignment [94] | Define changes in sodium content of fast food items at six QSR chain.  *Oceania:* Australia | 2009-2012  Descriptive  cross-sectional | **Outcomes (n=2)**  Sodium (mg)  Sodium density  (mg/100 g and mg/serving)  **Assessment/evidence**  Nutrient content data obtained from surveys of info on company websites for menu items (n=302 to 381 annually). Surveys were conducted in March annually 2009- 2012.  Data analyzed using Stata v 12.1.  **Guidelines/criteria**  Not reported | **6 QSR chains**  Domino’s  Hungry Jack’s *(Burger King)*  KFC  McDonald’s Subway  Pizza Hut | The mean sodium content of QSR products showed a modest decrease by 43 mg/100 g (95% CI, − 66 to − 20 mg/100 g) and 514 mg/100 g in 2009 to 471 mg/100 g in 2012.  Mean sodium content per serving was not significantly different at 654 mg in 2009 and 605 mg in 2012, reflecting wide variation in the serving sizes of items offered annually.  A small decline in sodium content was observed over four years across most food categories and by QSR chain, but many products still contain high levels of sodium. |
| Garemo and Naimi, 2018 [^[[18]](#endnote-18)^]  Text alignment [95] | Assess the dietary quality of children’s meals in restaurants by food groups and fried foods.  *Middle East:*  Abu Dhabi, United Arab Emirates | 2016  Descriptive  cross-sectional | **Outcomes (n=3)**  Energy (kcal)  Fat (g)  Sugar (g)  **Assessment/evidence**  Popular food outlets were identified using an online customer rating application. Menus were collected, and the meal quality was assessed for deep-frying and food group content.  **Guidelines/criteria**  US NRA’s Kids Live Well Program criteria | **58 restaurants**  Not reported  Combination of independent non-chain and transnational QSR and FSR chains, shopping malls eateries, and hotel restaurants | Half of restaurants (50%; 29/58) offered children’s menus that sold 209 meals, of which 60% were bundled and included beverages, but only 13% offered water or milk as default beverage.  More than three quarters (78.9%) of meals did not meet the US NRA’s Kids’ Live Well Program criteria, and nearly half of meals (46%; n=96/209) were deep-fried. |
| Hearst et al. 2013 [^[[19]](#endnote-19)^]  Text alignment [96] | Assess trends in nutritional quality of menu offerings at QSR chains.  *North America:* USA | Data examined in seven 2-year periods, of which five were relevant to our study’s  time frame:  2001–2002  2003–2004  2005–2006  2007–2008  2009– 2010  Descriptive  cross-sectional study | **Outcomes (n=3)**  Energy (kcal)  Sodium (g)  Saturated fat (g)  **Assessment/evidence**  Data for menu items and food and nutrient composition were obtained in 2011 from archived versions of the University of Minnesota Nutrition Coordinating Center Food and Nutrient Database for eight QSR chains.  **Guidelines/criteria**  HEI 2005 scores were calculated for each menu based on the extent that menu offerings were consistent with DGA 2005 | **8 QSR chains**  McDonald’s Burger King  Wendy’s  Taco Bell  KFC  Arby’s  Jack in the Box  Dairy Queen | A HEI 2005 score was assigned across all eight QSR chains that ranged from 45/100 in 1997/1998 to 48 in 2009/2010. Each individual QSR chain score ranged from 37 to 56 in 1997/1998, and 38 to 56 2009/2010.  Overall, the nutritional quality of menu offerings was poor. The most substantial improvements in nutritional quality were observed for meat/beans, and a decrease in saturated fat, and the proportion of calories from solid fats and added sugar.  The HEI 2005 score improved modestly (45-48) at six chains (i.e., McDonald’s, Taco Bell, KFC, Arby’s, Jack in the Box, and Dairy Queen) and decreased at two chains (i.e., Wendy’s and Burger King). |
| Heredia-Blonval et al. 2014 [^[[20]](#endnote-20)^]  Text alignment [97] | Examine the energy and salt content of products sold at QSR chains.  *Latin America and the Caribbean:* Costa Rica | Jan 2013  Descriptive  cross-sectional | **Outcomes (n=3)**  Energy (kcal)  Salt (mg)  Sodium density  (mg/100 g and mg/serving)  **Assessment/evidence**  Nutrient content assessed for products (n=311) across 10 food categories obtained from websites.  Mean salt content was compared between QSR chains and food categories.  **Guidelines/criteria**  Not reported | **7 QSR chains**  Domino’s  KFC  Pizza Hut  Popeye’s  Subway  Taco Bell  Teriyaki | Statistically significant differences were observed between the mean salt content across the seven QSR chains.  Subway’s products had the lowest mean salt content (0.97 g/100 g; p < 0.05). Popeye's and KFC had the highest mean salt content (1.57 g/100 g; p < 0.05). Significant variations in mean salt content were observed between food categories.  Salads had a mean salt content of 0.45 g/100 g while sauces had 2.16 g/100 g (p < 0.05). There was wide variation in salt content observed within food categories. Salt content in sandwiches ranged from 0.5 to 2.1 g/100 g. |
| Hobin et al. 2014 [^[[21]](#endnote-21)^]  Text alignment [98] | Compare the energy (calories), total fat and saturated fat, and sodium levels for the children’s menu items offered by four QSR chains across five countries.  *Europe:* United Kingdom  *North America:* Canada and USA  *Oceania:* Australia and  New Zealand | Aug 2012  Descriptive  cross-sectional | **Outcomes (n=5)**  Energy (kcal)  Fat (g)  Saturated fat (g)  Sodium (mg)  Serving size (g)  **Assessment/evidence**  Content analysis of menus for children’s meals (n=138) based on data obtained from websites or phone calls to companies in each country.  **Guidelines/criteria**  Not reported | **4 QSR chains**  Burger King (Hungry Jack’s)  KFC  McDonald’s  Subway | Results showed variation across the QSR chains and five countries for children’s menu items for energy, fat, saturated fat and sodium.  US chains had lower energy and portion sizes, and UK had lower sodium, respectively, compared to other countries (i.e., Australia, Canada and New Zealand, US).  Subway offered lower fat items compared to Burger King and KFC. Items offered at KFC were lower in saturated fat compared to Burger King. |
| Jacobson et al. 2013 [^[[22]](#endnote-22)^]  Text alignment [99] | Compare the mean levels of sodium for identical products in 2005, 2008, and 2011.  *North America:* USA | 2005-2011  Descriptive longitudinal | **Outcomes (n=1)**  Sodium (g)  **Assessment/evidence**  Restaurant website data compared to the DGA 2010 and computed for each period the mean (95% CI) sodium level per 100 g of product. The number and percentage of foods that had changed sodium levels that represented increases of at least 5% or at least 30% or did not change were identified.  **Guidelines/criteria**  DGA 2010 and American Heart Association (AHA) guidelines for high-risk populations to consume ≤ 1500 mg sodium/day | **16 QSR and FCR chains**  Arby’s  Au Bon Pain Blimpie  Burger King Chick-fil-A Domino’s Pizza Hardee’s  Jack in the Box KFC Little Caesars Pizza McDonald’s  Panera Bread  Papa John’s Pizza  Pizza Hut  Subway  Wendy’s | Between 2005 and 2011, the sodium content of 78 QSR products increased by 2.6%. Although some products showed decreases of at least 30%, a greater number of products increased at least 30%. There was no statistically significant change in sodium content over six years. |
| Jarlenski et al. 2016 [^[[23]](#endnote-23)^]  Text alignment [100] | Assessed changes in macronutrient profiles of items sold by QSR chains.  *North America:* USA | 2012 to 2014  Descriptive longitudinal | **Outcomes (n=4)**  Energy (kcal)  Fat (g)  Saturated fat (g)  Sugar (g)    **Assessment/evidence**  Data collected from MenuStat Database  2012-2014 (n=11,737 items) at 37 chains. Generalized linear models were used to examine differences in the macronutrient composition of newly introduced menu items.  **Guidelines/criteria**  Not reported | **37 QSR and FCR chains**  Not specified | From 2012 to 2014, only a minor decline in the calorie content (22-25 calorie reduction) was observed across 11,737 menu items assessed for changes in macronutrient composition. Over the period reviewed, beverages increased by 46 calories, newly introduced main course items reduced by 59 calories, and newly introduced dessert items increased by 90 calories, of which 57 calories were from added sugar. |
| Khan et al. 2018 [^[[24]](#endnote-24)^]  Text alignment [101] | Examine calories and sodium in menu items sold by QSR chains in four countries.  *North America:* USA  *Oceania:*  Australia  *Africa:*  Egypt  *Asia:*  India | Jul 2015  Descriptive  cross-sectional | **Outcomes (n=2)**  Energy (kcal)  Sodium density  (g/100 g)  **Assessment/evidence**  All menu items and food ingredients were taken from the food labels publicly listed by QSR chains through print or electronic media.    **Guidelines/criteria**  USDA and DGA 2015 recommendations for dietary sodium ≤ 2,300 mg/day for adults and  ≤ 1500 mg/day for children and adults > 50 years | **3 QSR chains**  McDonald’s  KFC  Pizza Hut | The energy content of KFC items (1,028 kcal) and McDonald’s items (896 kcal) were highest in Egypt. The Big Mac at McDonald’s in the US and Australia had the highest energy (530 and 493 kcal/serving), respectively, which represented ~22–24% of the daily calorie target of 2,200 kcal daily. Sodium content for the items in Arabia, US and Australia were 1,080 mg, 960 mg, and 859 mg, respectively, representing 41.7% and 37.3% of the recommended daily sodium intake for 8–50 year olds; and 47%, 64%, and 57%, respectively, for children below 8 years and adults older than 50 years based on 1,500 mg limit.  Different brands of similar foods had different sodium content. Two thirds (66.5%) of sodium came from meats, chicken and buns. |
| Kirkpatrick et al. 2013 [^[[25]](#endnote-25)^]  Text alignment [102] | Evaluate children’s menu items at five QSR chains compared to US dietary guidelines.  *North America:*  USA | 2008-2009 Descriptive cross-sectional | **Outcomes (n=5)**  Energy (kcal) Energy from fat (%) Energy from added sugar (%) Saturated fat (g) Sodium (g)  **Assessment/evidence**  Data collected from the restaurant database. Recommendations vary in relation to energy requirements, scores for all components of the HEI 2005 were calculated (eg, amount per 4184 kJ/1000 kcal) rather than using absolute amounts of foods or nutrients.  Restaurant websites menus were coded using Food and Nutrient Database for Dietary Studies and HEI 2005 score.  **Guidelines/criteria**  DGA 2005 and HEI 2005 | **5 QSR chains**  Burger King McDonald’s Subway  Taco Bell  Wendy’s | Full menus at QSR chains scored lower than 50/100 points on the HEI-2005. Children’s menus scored 10 points higher on average, and items marketed as healthy or nutritious scored 17 points higher compared to full menus. No menu or subset of menu items received a score higher than 72 out of 100 points. Scores for total fruit, whole grains and sodium were poor. |
| Mazariegos et al. 2016 [^[[26]](#endnote-26)^]  Text alignment [103] | Compare the nutritional quality of children’s combination meals with and without health claims.  *Latin America and the Caribbean:* Guatemala | 2016  Descriptive  cross-sectional | **Outcomes (n=6)**  Energy (kcal)  Sodium (mg)  Sugar (g)  TFA (g)  Saturated fat (%)  Energy from fat (%)  **Assessment/evidence**  Nutrition information requested at the point of sale from the restaurant manager, checking the restaurant website, or calling customer service. Combo meals classified as “healthy” or “less healthy” using the UK Nutrient Profiling Model. REDCap was used for data entry and STATA v 13.0.  **Guidelines/criteria**  NAM/USDA’s NSLP standards and UK’s Nutrient Profiling Model for children | **6 QSR chains**  McDonald’s Burger King Wendy’s  Pollo Campero  KFC  Pizza Hut | Of 114 combo meals, 21 (18.4%) were marketed for children. Only five meals (24%) provided nutrition information, and all were classified as “less healthy.”  Nutrient content for selected Guatemalan children’s combo meals were:  Median Range  energy (kcal) 514 (404-725)  sodium (mg) 885 (495-1173)  sugar (g) 46 (36-52)  sat fat (%) 11 (8-13)  energy from fat (%) (5) 39 (23-52)  TFA (g) 0 (0-0) |
| Moran et al. 2017 [^[[27]](#endnote-27)^]  Text alignment [104] | Examine the trends in nutrient content of children’s menus at US restaurant chains over three years.  *North America:* USA | 2012-2015  Descriptive  cross-sectional | **Outcomes (n=3)**  Energy (kcal)  Sodium (mg)  Saturated fat (g)  **Assessment/evidence**  Nutrients in children’s menu items (n=4,016) from 45 chains were extracted from MenuStat Database. Bootstrapped mixed linear models estimated changes in mean calories, saturated fat, and sodium in children’s food and beverage menu items between 2012 and 2013, 2014, and 2015. Changes in nutrient content of these items over time were compared to restaurants participating in the US NRA’s Kids Live Well Program criteria and non-participating restaurants. Data analyzed in 2016.  **Guidelines/criteria**  US NRA’s Kids Live Well  Program | **45 QSR, FCR and FSR chains**  Applebee’s Subway  Chipotle  Arby’s  Panera Bread Wendy’s  Burger King | From 2012 to 2014, calories in beverages offered with children’s menus increased by 11 calories.  From 2012 to 2015, no significant changes were  observed for calories in six FCR beverages, total calories, sodium or saturated fat in children’s menu offerings.  Restaurants that participated in the US NRA’s Kids Live Well program (n=15) had significantly reduced children’s entrée calories between 2012 and 2014 (by 40 calories/meal) compared to nonparticipating restaurants, but this change did not persist for the 2012 to 2015 period. |
| O’Donnell et al. 2008 [^[[28]](#endnote-28)^]  Text alignment [105] | Assess the nutrient quality of children’s meals at QSR chains.  *North America:*  Houston, TX, USA | Jul 2007    Descriptive  cross-sectional | **Outcomes (n=6)**  Energy (kcal)  Fat (g) Energy from fat (%) Saturated fat (g)  Sugar (g) Sodium (mg)  **Assessment/evidence**  Nutrition information was collected via phone calls to restaurant chains. Data analyzed with SAS.  **Guidelines/criteria**  NAM/USDA’s NSLP nutrition standards | **10 QSR chains**  Arby’s  Burger King Chick-fil-A  KFC  McDonald’s  Sonic  Subway  Taco Bell  Wendy’s Whataburger | Only 3% of children’s meals met all NSLP criteria. The meals that met all criteria offered a side of fruit plus milk, and most were deli-sandwich meals.  Meals that met the criteria had about one-third fat, one-sixth added sugar, twice the iron, and three times the amount of vitamin A and calcium compared to meals that did not meet the criteria.  Meals that did not meet the NSLP criteria were more than 1.5 times more energy dense than those that did meet the criteria. |
| Prentice et al. 2015 [^[[29]](#endnote-29)^]  Text alignment [106] | Examine the sodium content of food items at QSR chains and independent outlets to estimate the contribution of sodium to the diet of the New Zealand population using the 2008/09 New Zealand Adult Nutrition Survey  *Oceania:* New Zealand | 2008-2009  Descriptive  cross-sectional | **Outcomes (n=1)**  Sodium (mg)  **Assessment/evidence**  Nutrient analysis was conducted for the sodium content of savory foods from QSR chains (n=471). Nutrition information obtained from company websites. Nutrient content of 12 most popular foods from independent outlets (n=52) across 8 chains was determined using laboratory analysis.  **Guidelines/criteria**  UK FSA’s 2012 sodium targets | **8 QSR chains**  McDonald’s  Burger King  KFC  Domino’s  Hell’s Pizza  Pizza Hut  Subway  Wendy’s | Twelve out of thirteen of the QSR food categories exceeded the UK FSA’s 2012 sodium targets.  Sauces/salad dressings and fried chicken had the highest sodium content (per 100g) and from independent outlets, sausage rolls, battered hotdogs and mince and cheese pies were highest in sodium (per 100g). The mean daily sodium intake from savory fast foods was 283mg/d for the total adult population and 1229 mg/day for QSR consumers. |
| Reeves et al. 2011 [^[[30]](#endnote-30)^]  Text alignment [107] | Investigate the nutritional content and portion size children’s meals at QSR and non-chain FSR.  *Europe:* London, England,  UK | Jul and Aug 2009  Descriptive  cross-sectional | **Outcomes (n=4)**  Energy (kcal)  Fat (g)  Sodium (mg)  Portion size (g)  **Assessment/evidence**  Nutrient analysis was compared to standards, and data collected by online websites and at  restaurants. Chi-square tests compared the availability of nutrition information of fast food and table service restaurants.  **Guidelines/criteria**  Nutrient standards for children aged 5-11 years based on the UK Caroline Walker Trust guidelines | **7 QSR chains and 15 non-chain FSR**  Not specified | Mean portion size was significantly smaller in QSR chains (220.83 ± 65 g) compared to non-chain FSR (350.40 ± 110 g). Neither the QSR nor FSR meals met the recommended nutrient standards for lunch for children aged 5-11 years. |
| Roberts et al. 2018 [^[[31]](#endnote-31)^]  Text alignment [108] | Measure the energy content of frequently ordered  QSR and FSR chain meals in six countries.  *Africa:*  Accra, Ghana  *Americas:*  Boston, MA, USA and Ribeirao Preto, Brazil  *Asia:*  Beijing, China and Bangalore, India  *Europe:*  Kuopio, Finland | 2014 and 2017  Descriptive  cross-sectional | **Outcomes (n=2)**  Energy (kcal)  Energy density (kcal/g)  **Assessment/evidence**  Data collected from internet searches, site visits, and lab analysis of selected items using bomb calorimetry. Differences were calculated using least squares means and 95% confidence intervals.  **Guidelines/criteria**  2000 kcal/meal daily energy requirement for an adult woman | **111 QSR and**  **FSR chains**  Not reported | Weighted mean energy of restaurant meals was lower only in China (719 [95% CI 646 to 799] kcal versus 1088 [1002 to 1181] kcal; P <0.001).  The country, restaurant type, number of meal components, and meal weight predicted meal energy. A majority (94%) of FSR meals and 72% of QSR meals contained at least 600 kcal. QSR meals contained 33% less energy than FSR meals.  Excluding China, consuming QSR and FSR meals daily would provide between 70% and 120% of the daily energy requirement for a sedentary woman. |
| Rudelt et al. 2014 [^[[32]](#endnote-32)^]  Text alignment [109] | Examine trends in the sodium content of menu offerings at  eight QSR chains over 14 years.  *North America:* USA | 1997/1998 and 2009/2010  Descriptive  cross-sectional | **Outcomes (n=1)**  Sodium (mg)  **Assessment/evidence**  Percentage change in mean sodium (mg)/menu item was calculated between these two time periods.  Menu offerings and nutrient composition information for the menu items were obtained from archival versions of the University of Minnesota Nutrition Coordinating Center (NCC) Food and Nutrient Database. Nutrient composition information for lunch/dinner menu items sold by the QSR chains was updated in the database biannually. Menus were analyzed for changes in mean sodium content of all menu offerings except beverages, and specific categories of menu items among all restaurants and for each individual restaurant.  **Guidelines/criteria**  Maximum intake of  ≤ 2,300 mg sodium/day | **8 QSR chains**  McDonald’s  Burger King  Wendy’s  Taco Bell  KFC  Arby’s  Jack in the Box  Dairy Queen | No restaurant chain had reduced the sodium content across the lunch/dinner menu offerings over 14 years (including 2000 – 2010). The mean sodium content of menu offerings across the eight chains increased by 23·4 %. The mean sodium content of entrées increased by 17·2% and condiments increased by 26·1 %. Only side dishes showed a decrease of sodium by 6·6 %. |
| Schoffman et al. 2016 [^[[33]](#endnote-33)^]  Text alignment [110] | Determine and compare the energy content of entrees sold at QSR versus FCR chains.  *North America:* USA | Jan 2014  Descriptive  cross-sectional | **Outcomes (n=1)**  Energy (kcal)  **Assessment/evidence**  Data collected from the MenuStat Database 2014.  Mean energy (kcal) per entrée between QSR and FCR, and the proportion of restaurant entrées that fell into different calorie ranges were assessed based on a statistical significance of P<0.05.  **Guidelines/criteria**  Not reported | **62 QSR and FCR chains**  White Castle  Panda Express  Krystal  Steak ’N Shake  Subway  Einstein Brothers  Wienerschnitzel  Bruegger’s Bagels  Taco Bell  Five Guys  In-N-Out Burger  Au Bon Pain  A&W  Panera Bread  Del Taco  Noodles & Company  McDonald’s  Cosi  Chick-Fil-A  Qdoba  Taco Bueno  Schlotzsky’s  Arby’s  Potbelly’s Sandwich Works  Taco John’s  Chipotle  Burger King  Corner Bakery Cafe  Hardee’s  Pollo Tropical  Wendy’s  Culver’s  Charley’s Grilled Subs  McAlister’s Deli  Tropical Smoothie Cafe  Jason’s Deli  Jack in the Box  Moe’s Southwest Grill  Dairy Queen  Smashburger  KFC  Dickey’s Barbecue Pit  El Pollo Loco  Togo’s Eatery/Sandwiches  Checker’s Drive-In/Rally’s  Baja Fresh  Sonic  Zaxby’s  Church’s Chicken  Firehouse Subs  Carl’s Jr.  Captain D’s  Fazoli’s  Pei Wei  Quiznos  Boston Market  Jimmy John’s  Whataburger  Bojangles’  Popeyes  Taco Cabana  Long John Silver’s | A total of 3,193 entrées were analyzed at 34 QSR and 28 FCR chains. FCR chains provided significantly more calories per entrée (760 kcal) than QSR entrées (561 kcal). QSRs provided significantly more entrées in the lower calorie categories (< 500 calories/item) and FCRs provided more entrées in the higher-calorie categories (> 751 calories/item). |
| Scourboutakos and  L'Abbé, 2012 [^[[34]](#endnote-34)^]  Text alignment [111] | Analyze the calorie content of restaurant food items to determine factors that may influence the effectiveness of menu calorie labeling.  *North America:* Canada | Sept-Dec 2010  Descriptive  cross-sectional | **Outcomes (n=3)**  Energy (kcal)  Energy density  (% kcal/100g food)  portion size (g)  **Assessment/evidence**  Nutrition information was collected from websites of chain restaurants for n=4178 side dishes, entrees, and individual items at 85 chains.  Data analyzed in 2011 using statistical analysis (p= <0.05) considered significant for mean serving size, calories, and calorie density.  **Guidelines/criteria**  Not reported | **85 QSR and FSR chains**  *Only few names were mentioned*  Boston Rouge  Boston Pizza  Casey's  Denny's  Earl's Restaurant  East Side Mario's  Jack Astors  Joey's Restaurant  Kelsey's  Mike's Restaurant  Milestone's  Montana's  Mr. Greek  Pizza Delight  Pizza Hut  Scores Rotisserie  Shoeless Joe's  Swiss Chalet  The Keg  White Spot | FSR chains had higher calories/serving for all food categories compared to QSR chains. There was substantial variation in calories both within and across food categories. Serving size was more strongly correlated with calories than caloric density. Higher-calorie items had a larger serving size compared to lower-calorie items, but did not differ significantly by calorie density. |
| Scourboutakos et al. 2013 [^[[35]](#endnote-35)^]  Text alignment [112] | Analyze the nutritional profile of breakfast, lunch, and dinner meals from FSR chains.  *North America:* Canada | 2010-2011  Descriptive  cross-sectional | **Outcomes (n=5)**  Energy (kcal)  Fat (g)  Saturated fat (g)  TFA (g)  Sodium (mg)  **Assessment/evidence**  Total of 3,507 different variations of 685 meals and 156 desserts.  Nutrition information collected from online websites. Nutrient values calculated as a percentage of the daily value (%DV).  **Guidelines/criteria**  Daily Value (% DV) based on 2000 kcal/day and % Adequate Intake (AI) of sodium for adults; and NAM=1500 mg sodium/day | **19 FSR chains**  Not reported | Of 19 FSR chains, breakfast, lunch, and dinner meals consisted of 1128 calories (56% of the daily 2000 calorie recommendation), 151% of the amount of sodium (2269 mg), 89% of the DV for fat (58 g), 83% of the DV for saturated fat, and 0.6 g TFA.  More than 80% of meals exceeded the daily AI for sodium (1500 mg) and more than 50% exceeded the daily UL for sodium (2300 mg). Only 1% of meals had less than recommended target of 600 mg sodium/meal. Almost 50% of meals exceeded the DV for fat (65 g) and 25% exceeded the DV for saturated fat.  Restaurants labeled meals as “healthy” if they contained an average 474 calories, 13 g fat (20% DV), 3 g saturated fat (17% DV), and 752 mg of sodium (50% AI). |
| Scourboutakos et al. 2014 [^[[36]](#endnote-36)^]  Text alignment [113] | Analyze the added sugar content in children’s meals at QSR and FSR chains.  *North America:* Toronto, Canada | 2010  Descriptive  cross-sectional | **Outcomes (n=2)**  Total sugar (g)  Added sugar (g)  **Assessment/evidence**  Total sugar levels were taken from websites of 10 QSR and 7 FSR chains. Added sugar levels in children’s meals (n=3,178) were calculated in 2014 by subtracting all naturally occurring sugar from the total sugar level.  **Guidelines/criteria**  1800 kcal/day recommended by Canadian government for a 4 to 8 year old child; and the WHO guidelines for percentage energy from added sugar (5-10%/day) | **17 QSR and FSR chains**  Not specified | There was a wide range of added sugar in children’s meals ranging from 0 g to 114 g.  Half (50%) of children’s meals sold at chain restaurants exceeded the WHO's daily added sugar recommendation. |
| Scourboutakos and  L'Abbé, 2013 [^[[37]](#endnote-37)^]  Text alignment [114] | Evaluate the sodium levels in menu items for adults and children at QSR and FSR chains.  *North America:* Canada | Sept-Dec 2010  cross-sectional | **Outcomes (n=1)**  Sodium (mg)  **Assessment/evidence**  Nutrition information for 4,044 menu items was collected from FSR (n=20) and QSR (n=65) chain websites and entered into a database. Sodium content of products was compared to guidelines.  **Guidelines/criteria**  AI of sodium for adults = 1500 mg sodium/day and children = 1200 mg sodium/day  Upper Level (UL) for sodium = 2300 mg sodium/day  2012 and 2014 US National Sodium Reduction Initiative (NSRI) targets | **65 QSR and 20 FSR chains**  Not reported | Menu items at FSR chains contained 1,455 mg sodium/serving (or 97% of AI level of 1500 mg/day). At FSR chains, 40% of menu items exceeded AI for sodium and more than 22% of stir fry entrées, sandwiches/wraps, ribs, and pasta entrées with meat/seafood exceeded the daily UL for sodium.  QSR meal items contained an average of 1,011 mg sodium (68% of the daily AI), while side dishes at QSR and FSR chains contained 736 mg (49%).  Children's meal items contained an average of 790 mg/serving (66% of the sodium AI for children of 1200 mg/day). A small number of children's items exceeded the daily UL.  More than half (52%) of restaurants exceeded the 2012 NSRI sodium targets and 69% exceeded the 2014 sodium targets. |
| Scourboutakos et al. 2018 [^[[38]](#endnote-38)^]  Text alignment [115]  *North America:* Canada | Assess whether salt substitutes and enhancers were associated with changes in sodium levels at chain restaurants. | 2010-2016  Descriptive longitudinal | **Outcomes (n=1)**  Sodium (mg)  **Assessment/evidence**  A longitudinal database (MENU-FLIP) containing nutrition information for Canadian chain restaurants with 20 or more locations nationally were created in 2010 and updated in 2013 and 2016.  Changes in sodium levels (per serving) and  prevalence of salt substitutes/enhancers in 222 foods from 12 of the QSR chains were compared across three time points. Data analyzed using SAS v 9.3 software.  **Guidelines/criteria**  Not reported | **12 QSR, FCR and FSR chains**  A&W  Arby’s  Burger King  Edo Japan  KFC  McDonald’s  Pizza Pizza  Subway  Taco Del Mar  Taco Time  Tim Hortons | Sixty-nine percent of foods contained a salt substitute/enhancer. Substitutes/enhancers were found in every restaurant chain (n = 12) for which ingredient data were available. The most common substitutes/enhancers were yeast extracts (in 30% of foods), calcium chloride (28%), monosodium glutamate (14%) and potassium chloride (12%).  Sodium levels in foods that contained substitutes/enhancers decreased significantly more (190 ± 42 mg/serving) over the study period than those in foods that did not contain a substitute/enhancer (40 ± 17 mg/serving, p < 0.001). |
| Scourboutakos et al. 2014 [^[[39]](#endnote-39)^]  Text alignment [116] | Measure changes in sodium content of chain restaurant items over three years.  *North America:* Canada | 2010-2013  Descriptive longitudinal | **Outcomes (n=4)**  Energy (kcal)  Sodium (mg)  Sodium density  (mg/100 g)  Serving size  **Assessment/evidence**  Data for the serving size, calorie and sodium level of 3878 foods were collected from restaurant websites.  χ2 test used to compare the percentage of entrées with sodium levels (mg/serving) greater than the recommended AI level (1500 mg) and UL (2300 mg) in 2010 and 2013.  Data analyzed using SAS  v 9.3.  **Guidelines/criteria**  DRV = AI for sodium (1500 mg/day) and Tolerable UL for sodium (2300 mg/day). | **61 QSR, FCR and FSR chains**  241 Pizza  A&W  Arby’s  Baton Rouge  Bento Nouveau  Boston Pizza  Burger King  Casey’s Bar and Grill  Coffee Time  Country Style  Dagwoods Sandwiches and Salads  Dairy Queen  Denny’s  Druxy’s Deli  Earl’s Restaurant  East Side Mario’s  Edo Japan  Extreme Pita  Flying Wedge Pizza  Harvey’s  Jack Astor’s  Joey’s Restaurants  Jugo Juice  Kelsey’s  KFC  Little Caesars  Manchu Wok  McDonald’s  Mikes  Mmmuffins  Montana’s  Mr. Greek 2  Mr. Sub  Mrs. Vanelli’s Fresh Italian Foods  New Orleans Pizza  New York Fries  Opa! Souvlaki of Greece  Orange Julius  Panago  Pita Pit  Pizza  Pizza Delight  Pizza Hut  Pizza Nova  Pizza Pizza  Pizzaville  Robin’s Donuts  Scores Rotisserie  Shoeless Joe’s  Subway  Swiss Chalet  Taco Bell  Taco Del Mar  Taco Time  Teriyaki Experience  The Great Canadian Bagel  Tim Hortons  Treats  Van Houtte’s Bistro  White Spot Legendary Restaurant  White Spot Triple O’s | Sodium levels (mg/serving) decreased in 30.1% of foods, increased in 16.3% of foods, and were unchanged in 53.6% of foods examined.  The prevalence and magnitude of change varied depending on the restaurant and food category.  Average change in foods with a decrease in sodium was –220 (standard deviation [SD] ± 303) mg/serving (a decline of 19% [SD ± 17%]), whereas the average change in foods with an increase in sodium was 251 (SD ± 349) mg/serving (a 44% [SD ± 104%] increase).  Overall, there was a small, yet significant, decrease in sodium per serving (–25 [SD ± 268] mg, p < 0.001). However, the percentage of foods exceeding the daily sodium adequate intake (1500 mg) and tolerable upper intake level (2300 mg) remained unchanged. |
| Sliwa et al. 2016 [^[[40]](#endnote-40)^]  Text alignment [117] | Compare the nutritional content of available children’s meal combinations in leading LSR chains with national recommendations.  *North America:* USA | May 2014  Descriptive  cross-sectional | **Outcomes (n=5)**  Energy (kcal)  Fat (g)  Saturated fat (g)  Sodium (mg)  Portion size (g/oz)  **Assessment/evidence**  Data collected from leading 10 FSR and LSR restaurants from 2013 rankings. Menu screenshots were captured from restaurant websites for child menus.  Children's meal combinations analyzed for calorie, fat, saturated fat, and sodium content and compared to several guidelines.  **Guidelines/criteria**  DGA 2010 and expert recommendations =  ≤ 600 kcal, < 35% kcal from fat, < 105 kcal from saturated fat and < 770 mg sodium/meal | **20 chains**  **10 QSR chains**  Arby’s  Burger King  Chik-Fil-A  Dairy Queen  Jack-in-the-Box  KFC  McDonald’s  Sonic  Subway  Wendy’s  **10 FCR or FSR chains**  Applebee’s  Buffalo Wild Wings  Chili’s  Denny’s  IHOP  Olive Garden  Outback Steakhouse  Red Lobster  Red Robin  TGI Friday’s | Majority of QSR (72%) and FSR (63%) meal combinations were 600 kcal. Only 31.9% of children’s meal combinations at QSR chains and 21.7% at FSR chains met all 4 nutrient criteria (≤ 600 kcal/meal, < 35% kcal from fat, < 105 kcal from saturated fat and < 770 mg of sodium).  In QSR and FCR or FSR segments, calorie target was met more frequently and the sodium target less often. |
| Soo et al. 2018 [^[[41]](#endnote-41)^]  Text alignment [118] | Examine the nutritional quality of menu items promoted at four QSR chains.  *North America:* USA | Jun 2010 and  Jul 2013  Descriptive  cross-sectional | **Outcomes (n=5)**  Energy (kcal)  Saturated fat (g)  Sugar (g)  Sodium (mg)  Portion size (g)  **Assessment/evidence**  Menu items pictured on signs and menu boards were recorded at 400 outlets of four QSR chains.  Nutrition scores were calculated using the UK Nutrient Profiling Index for items ranging from 0 (poorest nutritional quality) to 100 (highest nutritional quality). Changes in Nutrient Profiling Index scores and energy of promoted foods and beverages were analyzed using linear regression and found significant differences between 2010 and 2013 (P< 0.05).  **Guidelines/criteria**  UK Nutrient Profiling Index | **4 QSR chains**  McDonald's Burger King Wendy's  Taco Bell | Promoted foods and beverages on general menu boards and signs remained below the 'healthier' cut-off at both time points. On general menu boards, pictured items were modestly healthier from 2010 to 2013 at all chains except Taco Bell, where pictured items increased in energy.  Foods and beverages pictured on the kids' section showed the greatest nutritional improvements. Although promoted foods on general menu boards and signs improved in nutritional quality, beverages remained the same or were less healthy. |
| Stender et al. 2006 [^[[42]](#endnote-42)^]  Text alignment [119] | Analyze and compare TFA content of selected fast food items across 20 countries:  Austria  Czech Republic  Denmark  England  Hungary  Finland  France  Germany  Italy  Netherlands  Norway  Peru  Poland  Portugal  Russia  Scotland  Spain  South Africa  Sweden  USA | Nov 2004 and Sept 2005  Descriptive  cross-sectional | **Outcomes (n=1)**  TFA (g)  **Assessment/evidence**  Foods were homogenized and TFA content analyzed by capillary gas chromatography.  Results for fries and chicken nuggets were expressed as amounts/serving (i.e., 171 g of fries and 160 g of chicken).  **Guidelines/criteria**  WHO recommendation for countries to virtually eliminate artificial TFA in the food supply | **2 QSR chains**  McDonald’s  KFC | The TFA content varied from <1 g/serving in Denmark and Germany to 10 g in New York (McDonald’s) and 24 g in Hungary (KFC).  Fifty percent of the 43 servings contained more than 5 g TFA/serving. Amount of daily intake was associated with a 25 percent increase in the risk of CHD.  Cooking oil used for fries at McDonald’s outlets in the USA and Peru contained 23 percent and 24 percent TFA whereas oils used in many European countries contained only 10 percent TFA, some countries as low as 1 percent (Denmark) and 5 percent (Spain). At KFC, some values for TFA content were above 30 percent. |
| Uechi, 2018 [^[[43]](#endnote-43)^]  Text alignment [120] | Assess the nutritional quality of children’s meals sold at chain restaurants.  *Asia:* Japan | Oct-Nov 2017  Descriptive  cross-sectional | **Outcomes (n=4)**  Energy (kJ)  Sugar (g)  Fat (g)  Sodium (mg)  **Assessment/evidence**  Children’s meals (n=438) were assessed at 42 locations. Data collected from restaurants’ websites and the analysis used  SAS version 9.4 and P<0.05.  **Guidelines/criteria**  Japanese School Lunch Program standards for  energy (≤ 2218 kJ), fat (≤30% energy), salt (g)  6-7 years: 2218 kJ (530 kcal/meal)  12-14 years: 3431 kJ (820 kcal/meal) | **20 chain restaurants**  Not specified | More than half of restaurants had aligned with the nutrient standards of the Japanese School Lunch Program for energy. Overall, 58·9%, 40·6%, and 34·5% of the children’s meals met the energy (≤2218 kJ), fat (≤30% energy) and salt (<2 g) content, respectively. About 15·5% of children’ meals met the recommended energy, fat and salt standards.  ‘Japanese-style’ (restaurant-level characteristic) was associated with a decrease in the fat and an increase in the salt content. |
| Urban et al. 2014 [^[[44]](#endnote-44)^]  Text alignment [121] | Examine variability of popular food items at QSR chains over 18 years.  *North America:* USA | Period of interest:2000-2013 Descriptive  longitudinal | **Outcomes (n=4)**  Energy (kcal)  Sodium (mg)  Saturated fat (g)  TFA (g)  **Assessment/evidence**  Items selected were fries, cheeseburgers, grilled chicken sandwich, and soda.  Data collected using an archival website. Time trends assessed using simple linear regression models.  **Guidelines/criteria**  2000 kcal/day; 2300 mg and 1500 mg sodium/day | **3 QSR chains**  Not specified | Energy content per serving differed among chain restaurants for all menu items. Energy content of 56% of items decreased (β range, -0.1 to -5.8 kcal) and the content of 44% increased (β range, 0.6-10.6 kcal).  Sodium content of 18% of items significantly decreased (β range, -4.1 to -24.0 mg) and 33% increased (β range, 1.9-29.6 mg).  After 2009, saturated fat and TFA content was modest for fries. In 2013, energy content of a large-sized bundled meal (cheeseburger, fries and soda) represented 65% to 80% of a 2,000 kcal/day. Sodium content represented 63% to 91% of the 2300 mg/day and 97% to 39% of the 1500 mg/day. |
| Urban et al. 2014 [^[[45]](#endnote-45)^]  Text alignment [122] | Analyze nutrient content of frequently ordered items from three QSR chains.  *North America:* USA | 2000 -2013 Descriptive  longitudinal | **Outcomes (n=3)**  Sodium density (mg/1000 kcal)  Saturated fat (g/1000 kcal)  TFA (g/1000 kcal)  **Assessment/evidence**  Products sampled: fried potatoes (large fries), cheeseburgers (2-oz and 4-oz), and a grilled chicken sandwich. They used an archival website to obtain data. The amount of each nutrient per 1,000 kcal was calculated to determine product reformulation trends. Data analyzed using SAS version 9.3.  **Guidelines/criteria**  Not reported | **3 QSR chains**  Not reported | Sodium content per 1000 kcal differed widely among the three chains by food item, precluding generalizations across chains. During the 14-year period, sodium content per 1000 kcal for large fries remained high at all chains, although the range narrowed from 316-2,000 mg per 1000 kcal in 2000 to 700-1,420 mg per 1000 kcal in 2013.  Cheeseburgers were the main contributor of saturated fat, and there was little change in content per 1000 kcal for this item during the 14-year period. In contrast, there was a sharp decline in saturated fat and TFA of large fries per 1000 kcal. After 2009, the major contributor of TFA/1000 kcal was cheeseburgers; and TFA content of this item remained stable during the 14-year period. |
| Waterlander et al. 2014 [^[[46]](#endnote-46)^]  Text alignment [123] | Determine the mean nutrient content and contribution to recommended  daily intakes for energy, saturated fat, sugar, and sodium.  *Oceania:*  New Zealand | January 2014  Descriptive  cross-sectional study | **Outcomes (n=4)**  Energy (kcal)  Saturated fat (g)  Sugar (g)  Sodium (mg)  **Assessment/evidence**  Online survey completed for four QSR chains based on Children’s menu info from restaurant websites. The most popular QSR items were determined (n=104 NZ adults in Jan 2014) that examined reported QSR intake over past month. Nutrient content of QSR items determined using the 2013 version of Nutritrack.  **Guidelines/criteria**  RDI for adult men and women, respectively, for energy 13,300/9900 kJ; saturated fat 42.3g/31.5g;  sugar 117.4/87.4g; and sodium 2,300 mg/day. Additionally, the WHO guideline for free or added sugar intake (5% RDI for energy). | **4 QSR chains** McDonalds  KFC  Pizza Hut  Burger King | The most popular burger combo meals and pizza contributed between one-third and a half of the adult’s RDI for energy and nutrients.  Combo meals provided at least 94% of the RDI for sugar when applying the new WHO guideline (5% RDI). The mean range in sodium content of salads available at different chains was 133 (172) mg per serving at KFC to 967 (809) mg per serving at Burger King. |
| Wellard et al. 2012 [^[[47]](#endnote-47)^]  Text alignment [124] | Analyze the nutritional composition of children’s meals at six QSR chains.  *Oceania:* New South Wales, Australia | November 2010  Descriptive  cross-sectional study | **Outcomes (n=4)**  Energy (kJ)  Saturated fat (g)  Sugar (g)  Sodium (mg)  **Assessment/evidence**  Data of nutritional composition of children’s meals were surveyed from restaurant websites, and estimated recommended daily quantities of nutrients were calculated for a 4, 8 and 13-year-old child.  **Guidelines/criteria**  Nutrient Reference Values and the Dietary  Guidelines for Children and Adolescents in Australia for saturated fat ≤ 10% total energy and sugar < 20% total energy. | **6 QSR chains**  Chicken Treat  Hungry Jack’s  KFC  McDonald’s  Oporto  Red Rooster | Of 199 children’s meal combinations analyzed, each chain had a different number of meal combinations that varied from 3 to 144.  The mean nutritional composition for all children’s meals was 2229 kJ, 6.4 g saturated fat, 27.7 g sugar and 702 mg sodium per meal.  Only 16% and 22% of meals met the industry’s nutrient criteria for children aged 4–8 and 9–13 years, respectively. Seventy-two percent of QSR meals exceeded 30% of the daily energy recommendations for 4 year old children, and 90% of meals exceeded 30% of the upper limit for sodium for children aged 4–8. Some meals also exceeded the upper limit for sodium and daily saturated fat recommendations for children aged 4–8 years. |
| Wellard-Cole et al. 2018 [^[[48]](#endnote-48)^]  Text alignment [125] | Examine the energy content of Australian QSR food menu items over seven years, before and after the introduction of menu board labelling, to determine the impact of the introduction of the legislation.  *Oceania:* Australia, New South Wales | 2009 and 2015  Observational  cross-sectional | **Outcomes (n=2)**  Energy (kJ)  Energy density (kJ/100 g and kJ/serving)  **Assessment/evidence**  Menu items were collected from the QSR chain websites annually and analyzed for the median energy content/serving of standard menu items/100 g to assess changes over six years. Data analyzed using SAS v 9.3.  **Guidelines/criteria**  Not reported | **5 QSR chains**  Hungry Jack's  KFC  McDonald's  Oporto  Red Rooster | Certain QSR chains had menu item categories with significant increases in the energy content over seven years. Overall, there were no significant or systematic decrease in energy following the introduction of menu labelling (*P*=0·19 by +17 kJ/100 g, *P*=0·83 by +8 kJ/serving). Limited-time only items were significantly higher in median energy content per 100 g than standard menu items (+74 kJ/100 g, *P*=0·002). |
| Wolfson et al. 2018 [^[[49]](#endnote-49)^]  Text alignment [126] | Assessed trends in sodium content of menu items at chain restaurants.  *North America:* USA | 2012 and 2016  Descriptive  Cross-sectional | **Outcomes (n=1)**  Sodium (mg)  **Assessment/evidence**  Data from 21,557 menu items were analyzed from the MenuStat Database.  Generalized linear models were used to examine changes in calorie-adjusted, per-item sodium content of menu items offered in all and items offered in 2012 only compared with items newly introduced in 2013, 2014, 2015, and 2016.  **Guidelines/criteria**  DGA 2015-2020 target of  ≤ 2300 mg sodium/day | **66 QSR, FCR and FSR chains**  Restaurants not reported | Calorie-adjusted sodium content in newly introduced menu items declined by 104 mg from 2012 to 2016 (p<0.02). The magnitude and direction of changes varied by menu category and restaurant type. Sodium content for main-course items was high. Sodium declined by 83 mg in QSR chains, 19 mg in FCR chains, and 163 mg in FSR chains.  Sodium in appetizer and side items newly introduced in 2016 increased by 266 mg compared with items on the menu in 2012 only (p<0.01). Sodium in main courses newly introduced in 2016 declined by 124 mg compared with items on the menu in 2012 only (p=0.01), with the greatest decline, 207 mg (p=0.03), among salads. |
| Ziauddeen et al. 2015 [^[[50]](#endnote-50)^]  Text alignment [127] | Compare the nutritional composition of QSR products in 10 countries.  *Asia:* China and Japan  *Europe:* Germany, Netherlands, United Kingdom  *Mediterranean:* United Arab Emirates  *North America:* Canada and USA  *Oceania:* Australia and New Zealand | Jan- Mar 2012  Descriptive  cross-sectional | **Outcomes (n=3)**  Energy (kJ) Fat (g) Saturated fat (g)  **Assessment/evidence**  Data for 2961 food and beverage products were collected from QSR chains’ websites. A survey of the reported nutrient content and content per 100 g of items was completed across 10 countries. Data checked for distribution and medians and ranges were calculated with  SPSS v21.  **Guidelines/criteria**  Not reported | **5 QSR chains**  Burger King  (*Hungry Jack’s in Australia and New Zealand*)  KFC  McDonald’s  Pizza Hut  Subway | There was considerable variability in energy and fat content of QSR products across the 10 countries, reflecting variability for the portfolio of products and serving sizes. Differences in total energy between countries were noted for chicken dishes (649–1197 kJ/100 g) and sandwiches (552–1050 kJ/100 g). When comparing the same product between countries, variations were consistently observed in total energy and fat content (g/100 g), such as McDonald’s Chicken McNuggets with 12 g total fat/100 g in Germany compared with 21.1 g/100 g in New Zealand. |

**Abbreviations and Acronyms**

AI (Adequate Intake); Dietary Guidelines for Americans (DGA); Dietary Reference Value (DRV); Daily Value (DV); Food and Drug Administration (FDA); Food Standards Agency (FSA); Healthy Eating Index (HEI); grams (g); Kentucky Fried Chicken (KFC); kilocalories (kcal); kilojoules (kJ); milligrams (mg); fast-casual restaurants (FCR); full-service restaurants (FSR); limited-service restaurants (LSR); National Academy of Medicine (NAM); quick-service restaurants (QSR); National Health and Nutrition Examination Survey (NHANES); National Sodium Reduction Initiative (NSRI); Recommended Daily Intakes (RDIs); trans fatty acids (TFA); United Kingdom (UK); United States of America (USA); United States Department of Agriculture (USDA); Upper Level (UL); and What We Eat in America (WWEIA).

**References**

1. . Ahuja, J.K.; Wasswa-Kintu, S.; Haytowitz, D.B.; Daniel, M.; Thomas, R.; Showell, B.; Nickle, M.; Roseland, J.M.; Gunn, J.; Cogswell, M.; Pehrsson, P.R. Sodium content of popular commercially processed and restaurant foods in the United States. *Prev Med Rep.* 2015, *2,* 962–967. Available online: https://doi.org/10.1016/j.pmedr.2015.11.003 (accessed May 2019). [↑](#endnote-ref-1)
2. . Astiasarán, I.; Abella, E.; Gatta, G.; Ansorena, D. Margarines and fast-food french fries: Low content of trans fatty acids. *Nutrients.* 2017, *9*, 662. Available online: https://doi.org/10.3390/nu9070662 (accessed on 31 October 2018). [↑](#endnote-ref-2)
3. . Auchincloss, A.H.; Leonberg, B.L.; Glanz, K.; Bellitz, S.; Ricchezza, A.; Jervis, A. Nutritional value of meals at full-service restaurant chains. *J Nutr Educ Behav.* 2014, *46*, 75–81. Available online: http://dx.doi.org/10.1016/j.jneb.2013.10.008 (accessed May 2019). [↑](#endnote-ref-3)
4. . Bauer, K.W.; Hearst, M.O.; Earnest, A.A.; French, S.A.; Oakes, J.M.; Harnack, L.J. Energy content of U.S. fast-food restaurant offerings: 14-year trends. *Am J Prev Med.* 2012, *43,* 490–497. Available online: https://doi.org/10.1016/j.amepre.2012.06.033 (accessed May 2019). [↑](#endnote-ref-4)
5. . Bleich, S.N.; Wolfson, J.A.; Jarlenski, M.P. Calorie changes in chain restaurant menu items: Implications for obesity and evaluations of menu labeling. *Am J Prev Med.* 2015, 48, 70–75. Available online: http://dx.doi.org/10.1016/j.amepre.2014.08.026 (accessed May 2019). [↑](#endnote-ref-5)
6. . Bleich, S.N.; Wolfson, J.A.; Jarlenski, M.P. Calorie changes in large chain restaurants: declines in new menu items but room for improvement. *Am J Prev Med.* 2016, *50,* e1–e8. Available online: https://doi.org/10.1016/j.amepre.2015.05.007 (accessed on 31 October 2018). [↑](#endnote-ref-6)
7. . Bleich, S.N.; Wolfson, J.A.; Jarlenski, M.P. Calorie changes in large chain restaurants from 2008 to 2015. *Prev Med.* 2017, *100,* 112–116. Available online: https://doi.org/10.1016/j.ypmed.2017.04.004 (accessed May 2019). [↑](#endnote-ref-7)
8. . Brindal, E.; Mohr, P.; Wilson, C.; Wittert, G. Obesity and the effects of choice at a fast food restaurant. *Obesity Research & Clinical Practice.* 2008, *2*, 111–117. Available online: https://doi.org/10.1016/j.orcp.2008.03.004 (accessed May 2019). [↑](#endnote-ref-8)
9. . Bruemmer, B.; Krieger, J.; Saelens, B.E.; Chan, N. Energy, saturated fat, and sodium were lower in entrées at chain restaurants at 18 months compared with 6 months following the implementation of mandatory menu labeling regulation in King County, Washington. *J Assoc Nutr Diet.* 2012, *112,* 1169–1176. Available online: https://doi.org/10.1016/j.jand.2012.04.019 (accessed May 2019). [↑](#endnote-ref-9)
10. . Chand, A.; Eyles, H.; Ni Mhurchu, C. Availability and accessibility of healthier options and nutrition information at New Zealand fast food restaurants. *Appetite.* 2012, *58,* 227–233. Available online: https://doi.org/10.1016/j.appet.2011.10.006 (accessed May 2019). [↑](#endnote-ref-10)
11. . Cohen, D.A.; Lesser, L.I.; Wright, C.; Story, M.; Economos, C. Kid’s menu portion sizes: how much should children be served? *Nutr. Today.* 2016, *51,* 273–280. Available online: http://dx.doi.org/10.1097/NT.0000000000000179 (accessed May 2019). [↑](#endnote-ref-11)
12. . Deierlein, AL, Peat, K, Claudio L. Comparison of the nutrient content of children’s menu items at US restaurant chains, 2010–2014. *Nutrition Journal*. 2015, 14, 80. Available online: https://doi.org/10.1186/s12937-015-0066-4 (accessed May 2019). [↑](#endnote-ref-12)
13. . Dunford, E.; Webster, J.; Barzi, F.; Neal, B. Nutrient content of products served by leading Australian fast food chains. *Appetite.* 2010, 55, 484–489. Available online: https://doi.org/10.1016/j.appet.2010.08.015 (accessed May 2019). [↑](#endnote-ref-13)
14. . Dunford, E.; Webster, J.; Woodward, M.; Czernichow, S.; Yuan, W.L.; Jenner, K.; Ni Mhurchu, C.; Jacobson, M.; Campbell, N.; Neal, B. The variability of reported salt levels in fast foods across six countries: opportunities for salt reduction. *CMAJ*. 2012, *184*, 1023–1028. Available online: https://doi.org/10.1503/cmaj.111895 (accessed May 2019). [↑](#endnote-ref-14)
15. . Eissa, M.A.; Hearne, K.; Saavedra, N. Comparison of children’s menu items at full- and quick-service restaurants. *Southern Medical Journal.* 2018, *111*, 192–197. http://dx.doi.org/10.14423/SMJ.0000000000000793 (accessed May 2019). [↑](#endnote-ref-15)
16. . Eyles, H.; Jiang, Y.; Blakely, T.; Neal, B.; Crowley, J.; Cleghorn, C.; Ni Mhurchu, C. Five year trends in the serve size, energy, and sodium contents of New Zealand fast foods: 2012 to 2016. *Nutr J.* 2018, *17,* 65. Available online: https://doi.org/10.1186/s12937-018-0373-7 (accessed May 2019). [↑](#endnote-ref-16)
17. . Garcia, J.; Dunford, E.K.; Sundtrom, J.; Neal, B.C. Changes in the sodium content of leading Australian fast-food products between 2009 and 2012. *Med. J. Aust.* 2014, *200*, 340–344. Available online: https://www.mja.com.au/system/files/issues/200_06_070414/gar10049_fm.pdf (accessed May 2019). [↑](#endnote-ref-17)
18. . Garemo, M.; Naimi, A.A. Children’s meals at restaurants in Abu Dhabi, United Arab Emirates, have poor nutritional quality. *Mediterranean Journal of Nutrition and Metabolism*. 2018, *11*, 85–92. Available online: http://dx.doi.org/10.3233/MNM-17178 (accessed May 2019). [↑](#endnote-ref-18)
19. . Hearst, M.O.; Harnack, L.J.; Bauer, K.W.; Earnest, A.A.; French, S.A.; Michael Oakes, J. Nutritional quality at eight U.S. fast-food chains: 14-year trends. *Am J Prev Med.* 2013, *44,* 589–594. Available online: https://doi.org/10.1016/j.amepre.2013.01.028 (accessed May 2019). [↑](#endnote-ref-19)
20. . Heredia-Blonval, K.; Blanco-Metzler, A.; Montero-Campos, M.; Dunford, E.K. The salt content of products from popular fast-food chains in Costa Rica. *Appetite.* 2014, *83*, 173–177. Available online: https://doi.org/10.1016/j.appet.2014.08.027 (accessed May 2019). [↑](#endnote-ref-20)
21. . Hobin, E; White, C.; Li, Y.; Chiu, M.; O'Brien, M.F.; Hammond, D. Nutritional quality of food items on fast-food 'kids' menus': comparisons across countries and companies. *Public Health Nutr.*2014, *17,* 2263–2269. Available online: https://doi.org/10.1017/S1368980013002498 (accessed May 2019). [↑](#endnote-ref-21)
22. . Jacobson, M.F.; Havas, S.; McCarter, R. Changes in sodium levels in processed and restaurant foods, 2005 to 2011. *JAMA Intern Med.* 2013, *173,* 1285–1291. Available online: http://dx.doi.org/10.1001/jamainternmed.2013.6154 (accessed May 2019). [↑](#endnote-ref-22)
23. . Jarlenski, M.P.; Wolfson, J.A.; Bleich, S.N. Macronutrient composition of menu offerings in fast food restaurants in the U.S. *Am J Prev Med.* 2016, *51,* e91–e97. Available online: https://doi.org/10.1016/j.amepre.2016.03.023 (accessed May 2019). [↑](#endnote-ref-23)
24. . Khan, M.A.; Khan, M.M.; Abdelhafiz Gadelrab, R.M. Sodium content in fast foods: assessment of menu items in selected countries. *Journal of Foodservice Business Research.* 2018, *21*, 553-569. Available online: https://doi.org/10.1080/15378020.2018.1493894 (accessed May 2019). [↑](#endnote-ref-24)
25. . Kirkpatrick, S.I.; Reedy, J.; Kahle, L.L.; Harris, J.L.; Ohri-Vachaspati, P.; Krebs-Smith, S.M. Fast-food menu offerings vary in dietary quality, but are consistently poor. *Public Health Nutr.* 2014, *17,* 924–931. Available online: https://doi.org/10.1017/S1368980012005563 (accessed May 2019). [↑](#endnote-ref-25)
26. . Mazariegos, S.; Chacón, V.; Cole, A.; Barnoya, J. Nutritional quality and marketing strategies of fast food children’s combo meals in Guatemala. *BMC Obesity.* 2016, *3*, 52. Available online: https://doi.org/10.1186/s40608-016-0136-y (accessed May 2019). [↑](#endnote-ref-26)
27. . Moran, A.J.; Block, J.P.; Goshev, S.G.; Bleich, S.N.; Roberto, C.A. Trends in nutrient content of children’s menu items in U.S. chain restaurants. *Am J Prev Med.* 2017, *52,* 284–291. Available online: https://doi.org/10.1016/j.amepre.2016.11.007 (accessed on 31 October 2018). [↑](#endnote-ref-27)
28. . O’Donnell, S.I.; Hoerr, S.L.; Mendoza, J.A.; Tsuei Goh, E. Nutrient quality of fast food kids meals. *Am J Clin Nutr.* 2008, *88,* 1388–1395. Available online: http://dx.doi.org/10.3945/ajcn.2008.26197 (accessed May 2019). [↑](#endnote-ref-28)
29. . Prentice, C.A.; Smith, C.; McLean, R.M. Sodium in commonly consumed fast foods in New Zealand: a public health opportunity. *Public Health Nutr.* 2016, *19*, 958–966. Available online: https://doi.org/10.1017/S1368980015001731 (accessed May 2019). [↑](#endnote-ref-29)
30. . Reeves, S.; Wake, Y.; Zick, A. Nutrition labeling and portion size information on children's menus in fast-food and table-service chain restaurants in London, UK. *J Nutr Educ Behav.*2011, *43*, 543–547. Available online: https://doi.org/10.1016/j.jneb.2010.12.006 (accessed May 2019). [↑](#endnote-ref-30)
31. . Roberts, S.; Das, S.K.; Suen, V.M.M et al. Measured energy content of frequently purchased restaurant meals: multi-country cross sectional study. *BMJ.* 2018, *363*, k4864. Available online: https://doi.org/10.1136/bmj.k4864 (accessed May 2019). [↑](#endnote-ref-31)
32. . Rudelt, A.; French, S.; Harnack, L. Fourteen-year trends in sodium content of menu offerings at eight leading fast-food restaurants in the USA. *Public Health Nutr.* 2014, *17,* 1682–1688. Available online: https://doi.org/10.1017/S136898001300236X (accessed May 2019). [↑](#endnote-ref-32)
33. . Schoffman, D.E.; Davidson, C.R.; Hales, S.B.; Crimarco, A.E.; Dahl, A.A.; Turner-McGrievy, G.M. The fast-casual conundrum: Fast-casual restaurant entrees are higher in calories than fast food. *J Acad Nutr Diet.* 2016, *116,* 1606–1612. Available online: https://doi.org/10.1016/j.jand.2016.03.020 (accessed May 2019). [↑](#endnote-ref-33)
34. . Scourboutakos, M.J.; L'Abbé, M.R. Restaurant menus: calories, calorie density, and serving size. *Am J Prev Med*. 2012, *43*, 249–255. Available online: http://dx.doi.org/10.1016/j.amepre.2012.05.018 (accessed May 2019). [↑](#endnote-ref-34)
35. . Scourboutakos, M.J.; Semnani-Azad, Z.; L'Abbe, M.R. Restaurant meals: almost a full day's worth of calories, fats, and sodium. *JAMA Intern Med.* 2013, *173*, 1373–1374. Available online: http://dx.doi.org/10.1001/jamainternmed.2013.6159 (accessed May 2019).   [↑](#endnote-ref-35)
36. . Scourboutakos, M.J.; Semnani-Azad, Z.; L'Abbé, M.R. Added sugars in kids’ meals from chain restaurants. *Prev Med Rep*. 2016, *3*, 391–393. Available online: http://dx.doi.org/10.1016/j.pmedr.2014.11.003 (accessed May 2019). [↑](#endnote-ref-36)
37. . Scourboutakos, M.J.; L'Abbé, M.R. Sodium levels in fast-food and sit-down restaurants. *Can J Public Health*. 2013, *104*, 2–8. Available online: http://dx.doi.org/10.17269/cjph.104.3683 (accessed May 2019). [↑](#endnote-ref-37)
38. . Scourboutakos, M.J.; Murphy, S.A.; L'Abbé, M.R. Association between salt substitutes/enhancers and changes in sodium levels in fast-food restaurants: A cross-sectional analysis. *CMAJ*. 2018, *6,* E118–E125. Available online: https://www.ncbi.nlm.nih.gov/pubmed/29514800 (accessed on 20 September 2018). [↑](#endnote-ref-38)
39. . Scourboutakos MJ, L'Abbé MR. Changes in sodium levels in chain restaurant foods in Canada (2010–2013): A longitudinal study. *CMAJ*. 2014; 343–351. Available online: https://doi.org/10.1016/j.jneb.2010.12.006 (accessed May 2019). [↑](#endnote-ref-39)
40. . Sliwa, S.; Anzman-Frasca, S.; Lynskey, V.; Washburn, K.; Economos, C. Assessing the availability of healthier children’s meals at leading quick-serve and full-service restaurants. *J Nutr Educ Behav.* 2016, *48,* 242–249. Available online: https://doi.org/10.1016/j.jneb.2016.01.004 (accessed on 11 November 2018). [↑](#endnote-ref-40)
41. . Soo, J.; Harris, J.L.; Davison, K.K.; Williams, D.R.; Roberto, C.A. Changes in the nutritional quality of fast-food items marketed at restaurants, 2010 v. 2013. *Public Health Nutr.* 2018, *21*, 2117–2127. Available online: https://doi.org/10.1017/S1368980018000629 (accessed May 2019). [↑](#endnote-ref-41)
42. . Stender, S.; Dyerberg, J.; Astrup, A. High levels of industrially produced *trans* fat in popular fast foods. *N Engl J Med.* 2006, *354*, 1650–1652. Available online: http://dx.doi.org/10.1056/NEJMc052959 (accessed May 2019). [↑](#endnote-ref-42)
43. . Uechi, K. Nutritional quality of meals offered to children (kids’ meals) at chain restaurants in Japan. *Public Health Nutr.* 2018, *21*, 3101–3110. Available online: https://doi.org/10.1017/S1368980018001891 (accessed May 2019). [↑](#endnote-ref-43)
44. . Urban, L.E.; Roberts, S.B.; Fierstein, J.L.; Gary, C.E.; Lichtenstein, A.H. Temporal trends in fast-food restaurant energy, sodium, saturated fat, and trans fat content, United States, 1996–2013. *Prev Chronic Dis.* 2014, *11*, 140202. http://dx.doi.org/10.5888/pcd11.140202 (accessed May 2019). [↑](#endnote-ref-44)
45. . Urban, L.E.; Roberts, S.B.; Fierstein, J.L.; Gary, C.E.; Lichtenstein, A.H. Sodium, saturated fat, and trans fat content per 1,000 kilocalories: temporal trends in fast-food restaurants, United States, 2000-2013. *Prev Chronic Dis.* 2014, *11,* E228. Available online: http://dx.doi.org/10.5888/pcd11.140335 (accessed May 2019). [↑](#endnote-ref-45)
46. . Waterlander, W.E.; Eyles, H.; Whitworth, L. Healthiness of popular fast food items in New Zealand: Plenty of room for improvement. *NZ Med J.* 2014, *127*, 102–105. Available online: https://www.nzma.org.nz/journal/read-the-journal/all-issues/2010-2019/2014/vol-126-no-1392/letters-waterlander (accessed May 2019). [↑](#endnote-ref-46)
47. . Wellard, L.; Glasson, C.; Chapman, K. Fries or a fruit bag? Investigating the nutritional composition of fast food children’s meals. *Appetite.* 2012, *58*, 105–110. Available online: https://doi.org/10.1016/j.appet.2011.09.024 (accessed May 2019). [↑](#endnote-ref-47)
48. . Wellard-Cole, L.; Goldsbury, D.; Havill, M.; Hughes, C.; Watson, W.L.; Dunford, E.K.; Chapman, K. Monitoring the changes to the nutrient composition of fast foods following the introduction of menu labelling in New South Wales, Australia: an observational study. *Public Health Nutr*. 2018, *21*, 1194–1199. Available online: http://dx.doi.org/10.1017/S1368980017003706 (accessed May 2019). [↑](#endnote-ref-48)
49. . Wolfson, J.A.; Moran, A.J.; Jarlenski, M.P.; Bleich, S.N. Trends in sodium content of menu items in large chain restaurants in the U.S. *Am J Prev Med*. 2018, *54*, 28–36. Available online: https://doi.org/10.1016/j.amepre.2017.08.018 (accessed May 2019). [↑](#endnote-ref-49)
50. . Ziauddeen N, Fitt E, Edney L, Dunford E, Neal B, Jebb SA. Variability in the reported energy, total fat and saturated fat contents in fast-food products across ten countries. *Public Health Nutr.* 2015, *18*: 2962–2969. Available online: https://doi.org/10.1017/S1368980015000336 (accessed May 2019).

    Supplemental Table 3. Published studies of transnational restaurant chains to reformulate products and standardize portions to meet

    healthy dietary guidelines by geographic region and country, 2000-2018. *The citations below [78-127] correspond to the text in the manuscript.*

    | Lead author, year | Africa  Ghana (n=1)  Egypt (n=1)  South Africa (n=1) | Americas  *North America:* Canada (n=9) and USA (n=29)  *Latin America and Caribbean or South America:* Brazil (n=1), Costa Rica (n=1), Guatemala (n=1), and  Peru (n=1) | Asia  China (n=2), India (n=2) and Japan (n=2) | Europe  16 countries (i.e., France, Netherlands); Spain (n=2) and UK (n=5) | Middle East  United Arab Emirates (n=2) | Oceania  Australia (n=9) and New Zealand (n=7) |
    | --- | --- | --- | --- | --- | --- | --- |
    | Ahuja et al. 2015 [79] |  | *North America:*  USA |  |  |  |  |
    | Astiasarán et al. 2017 [80] |  |  |  | *Europe:*  Spain  Pamplona, Navarra |  |  |
    | Auchincloss et al. 2014 [81] |  | *North America:*  USA |  |  |  |  |
    | Bauer et al. 2012 [82] |  | *North America:*  USA |  |  |  |  |
    | Bleich et al. 2015 [83] |  | *North America:*  USA |  |  |  |  |
    | Bleich et al. 2016 [84] |  | *North America:*  USA |  |  |  |  |
    | Bleich et al. 2017 [85] |  | *North America:*  USA |  |  |  |  |
    | Brindal et al. 2008 [86] |  |  |  |  |  | *Oceania:* Australia |
    | Bruemmer et al. 2012 [87] |  | *North America:*  US |  |  |  |  |
    | Chand et al. 2012 [88] |  |  |  |  |  | *Oceania:*  New Zealand |
    | Cohen et al. 2017 [78] |  | *North America:*  USA |  |  |  |  |
    | Deierlein et al. 2015 [89] |  | *North America:*  USA |  |  |  |  |
    | Dunford et al. 2010 [90] |  |  |  |  |  | *Oceania:* Australia |
    | Dunford et al. 2012 [91]  *Six countries across three regions* |  | *North America:* Canada and USA |  | *Europe:* France and UK |  | *Oceania:* Australia and  New Zealand |
    | Eissa et al. 2017 [92] |  | *North America:* USA |  |  |  |  |
    | Eyles et al. 2018 [93] |  |  |  |  |  | *Oceania:*  New Zealand |
    | Garcia et al. 2014 [94] |  |  |  |  |  | *Oceania:* Australia |
    | Garemo and Naimi, 2018 [95] |  |  |  |  | *Middle East:*  Abu Dhabi, United Arab Emirates |  |
    | Hearst et al. 2013 [96] |  | *North America:* USA |  |  |  |  |
    | Heredia-Blonval et al. 2014 [97] |  | *Latin America:*  Costa Rica |  |  |  |  |
    | Hobin et al. 2014 [98]  *Five countries across three regions* |  | *North America:* Canada and USA |  | *Europe:* UK |  | *Oceania:* Australia and  New Zealand |
    | Jacobson et al. 2013 [99] |  | *North America:* USA |  |  |  |  |
    | Jarlenski et al. 2016 [100] |  | *North America:* USA |  |  |  |  |
    | Khan et al. 2018 [101]  *Four countries across four regions* | *Africa:*  Egypt | *North America*: USA | *Asia:*  India |  |  | *Oceania:* Australia |
    | Kirkpatrick et al. 2013 [102] |  | *North America:* USA |  |  |  |  |
    | Mazariegos et al. 2016 [103] |  | *Latin America:* Guatemala |  |  |  |  |
    | Moran et al. 2017 [104] |  | *North America:* USA |  |  |  |  |
    | O’Donnell et al. 2008 [105] |  | *North America:*  Houston, TX, USA |  |  |  |  |
    | Prentice et al. 2015 [106] |  |  |  |  |  | *Oceania:*  New Zealand |
    | Reeves et al. 2011 [107] |  |  |  | *Europe:* London, England, UK |  |  |
    | Roberts et al. 2018 [108]  *Six countries across four regions* | *Africa:*  Accra, Ghana | *North America:* Boston, USA  *South America:*  Ribeiro Preto, Brazil | *Asia:*  Beijing, China and  Bangalore, India | *Europe:* Kuopio, Finland |  |  |
    | Rudelt et al. 2014 [109] |  | *North America:* USA |  |  |  |  |
    | Schoffman et al. 2016 [110] |  | *North America:* USA |  |  |  |  |
    | Scourboutakos and L'Abbé, 2012 [111] |  | *North America:* Canada |  |  |  |  |
    | Scourboutakos et al. 2013 [112] |  | *North America:* Canada |  |  |  |  |
    | Scourboutakos et al. 2014 [113] |  | *North America:* Canada |  |  |  |  |
    | Scourboutakos and L'Abbé, 2013 [114] |  | *North America:* Canada |  |  |  |  |
    | Scourboutakos et al. 2016 [115] |  | *North America:* Canada |  |  |  |  |
    | Scourboutakos et al. 2014 [116] |  | *North America:* Canada |  |  |  |  |
    | Sliwa et al. 2016 [117] |  | *North America:* USA |  |  |  |  |
    | Soo et al. 2018 [118] |  | *North America:* USA |  |  |  |  |
    | Stender et al. 2006 [119]  *Three regions and 20 countries* | *Africa:*  South Africa | *North America:* USA  *South America:*  Peru |  | *Europe*  Austria  Czech Republic  Denmark  Hungary  Finland  France  Germany  Italy  Netherlands  Norway  Poland  Portugal  Russia  Spain  Sweden  United Kingdom (UK) [Aberdeen, Scotland;  London, England] |  |  |
    | Uechi, 2018 [120] |  |  | *Asia:* Japan |  |  |  |
    | Urban et al. 2014 [121] |  | *North America:* USA |  |  |  |  |
    | Urban et al. 2014 [122] |  | *North America:* USA |  |  |  |  |
    | Waterlander et al. 2014 [123] |  |  |  |  |  | *Oceania:*  New Zealand |
    | Wellard et al. 2012 [124] |  |  |  |  |  | *Oceania:* New South Wales, Australia |
    | Wellard-Cole et al. 2018 [125] |  |  |  |  |  | *Oceania:* New South Wales, Australia |
    | Wolfson et al. 2018 [126] |  | *North America:* USA |  |  |  |  |
    | Ziauddeen et al. 2015 [127]  *Ten countries across five regions* |  | *North America:* Canada and USA | *Asia:*  China and Japan | *Europe:* Germany, Netherlands, UK | *Mediterranean:* United Arab Emirates | *Oceania:* Australia and New Zealand |

    [↑](#endnote-ref-50)
